# Immune profiling of the macroenvironment in colorectal cancer unveils systemic dysfunction and plasticity of immune cells

**DOI:** 10.1002/ctm2.70175

**Published:** 2025-02-11

**Authors:** Haoxian Ke, Peisi Li, Zhihao Li, Xian Zeng, Chi Zhang, Shuzhen Luo, Xiaofang Chen, Xinlan Zhou, Shichen Dong, Shaopeng Chen, Junfeng Huang, Ming Yuan, Runfeng Yu, Shubiao Ye, Tuo Hu, Zhonghui Tang, Dongbin Liu, Kui Wu, Xianrui Wu, Ping Lan

**Affiliations:** ^1^ Guangdong Provincial Key Laboratory of Malignant Tumor Epigenetics and Gene Regulation, Department of Gastrointestinal Surgery, Sun Yat‐Sen Memorial Hospital Sun Yat‐Sen University Guangzhou China; ^2^ Guangdong Provincial Key Laboratory of Colorectal and Pelvic Floor Diseases The Sixth Affiliated Hospital of Sun Yat‐Sen University Guangzhou China; ^3^ Biomedical Innovation Center The Sixth Affiliated Hospital of Sun Yat‐Sen University Guangzhou China; ^4^ Department of Colorectal Surgery, Department of General Surgery The Sixth Affiliated Hospital, Sun Yat‐Sen University Guangzhou China; ^5^ Zhongshan School of Medicine Sun Yat‐Sen University Guangzhou China; ^6^ HIM‐BGI Omics Center, Hangzhou Institute of Medicine (HIM) Chinese Academy of Sciences, BGI Research Hangzhou China; ^7^ Guangdong Provincial Key Laboratory of Human Disease Genomics, Shenzhen Key Laboratory of Genomics BGI Research Shenzhen China; ^8^ BGI Genomics Harbin China; ^9^ College of Life Sciences University of Chinese Academy of Sciences Beijing China; ^10^ BGI Research Shenzhen China; ^11^ Shenzhen Key Laboratory of Single‐Cell Omics BGI Research Shenzhen China

**Keywords:** colorectal cancer, single‐cell omics, spatial transcription, tumour macroenvironment

## Abstract

**Background:**

Tumour immune macroenvironment is comprised of tumour and surrounding organs responding to tumourigenesis and immunotherapy. The lack of comprehensive analytical methods hinders its application for prediction of survival and treatment response in colorectal cancer (CRC) patients.

**Methods:**

Cytometry by time‐of‐flight (CyTOF) and RNA‐seq was applied to characterise immune cell heterogeneity in a discovery cohort including tumour, blood and intestinal architecture comprising epithelium, lamina propria, submucosa, muscularis propria of normal bowel and tumour–adjacent bowel tissues. Immunoprofiling was also validated by a validation cohort using single‐cell RNA sequencing, spatial transcription, CyTOF and multiplex immunofluorescent staining.

**Results:**

Based on cell phenotype and transcription, we identify distinct immunotypes in the CRC macroenvironment including blood, tumour and different intestinal architecture, showing disturbed immune cell compositions, increasing expression of immunosuppressive markers and cell–cell interactions contributing to immunosuppressive regulation. Furthermore, we evaluate immune macroenvironment influencing factors including tertiary lymphoid structures (TLSs), consensus molecular subtypes (CMSs) and immune checkpoint inhibitors (ICIs). TLS presence fuels anti‐tumour immunity by promoting CD8^+^ T cell infiltration and altering activation or suppression of T cell systematically. TLS presence correlates with patient survival, intrinsic CMS and therapeutic efficacy of ICI. PD‐1 and CD69 expressed in effector memory CD8^+^ T cells from blood can predict TLS presence in the CRC macroenvironment, serving as potential biomarkers for stratifying CRC patients into immunotherapy.

**Conclusions:**

Our findings provide insights into the CRC immune macroenvironment, highlighting immune cell suppression and activation in tumourigenesis. Our study illustrates the potential utility of blood for predicting immunotherapy response.

**Key points:**

- Distinct immunotypes are identified in the CRC macroenvironment.
- TLS and immunotherapy exert influence on the immune macroenvironment.
- TLS presence correlates with patient survival, CMS and therapeutic efficacy of ICI.
- PD‐1 and CD69 expressed in CD8+ Tem from blood can predict TLS presence in the CRC macroenvironment.

## Graphical abstract

1. Distinct immunotypes are identified in the CRC macroenvironment.
2. TLS and immunotherapy exert influence on the immune macroenvironment.
3. TLS presence correlates with patient survival, CMS and therapeutic efficacy of ICI.
4. PD‐1 and CD69 expressed in CD8+ Tem from blood can predict TLS presence in the CRC macroenvironment.

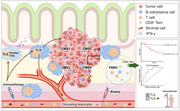

## 1 BACKGROUND

Colorectal cancer (CRC) has been one of the most common cancer with an estimated 1 926 136 cases in 2022 as well as a high mortality rate worldwide.^1^ The current therapies including surgery, chemotherapy and radiotherapy have significantly improved survival of CRC patients. However, although most patients with early‐stage CRC can be cured, high incidence of recurrences and distant metastasis was observed.^2^, ^3^, ^4^ In the past 10 years, neoadjuvant therapy based on immune checkpoint inhibitors (ICIs) has showed a promising therapeutic effect.^5^ Approximately 70%–80% of patient with mismatch repair‐deficient (dMMR) CRC showed pathological complete response (pCR) for ICI treatment.^6^ However, CRC is a heterogenous disease and biomarkers predictive of ICI response are not well elucidated.^7^, ^8^ Currently, microsatellite instability‐high (MSI‐H)/dMMR, PD‐L1 combined positive score (CPS) and tumour mutation burden (TMB) are confirmed to suggest greater efficacy of ICI treatment despite some limitations. MSI‐H contributed to accumulation of somatic mutation but only makes up of approximate 15% of CRC patients.^9^ Although PD‐L1 CPS has been adopted in clinical practice, it was reported that the survival benefit with ICI treatment was independent of PD‐L1 positivity.^10^, ^11^ Tumours with high TMB reveal positive treatment outcomes for ICI treatment.^12^, ^13^, ^14^ However, TMB is calculated by the total number of somatic alterations identified by genome sequencing, making a measurement hurdle. Tumour microenvironment (TME) is an ecosystem comprising immune cells, stromal cells and extracellular components which organises surrounding architecture and also determines tumour progression as well as therapeutic response. Recently, a considerable number of studies have unveiled TME heterogeneity and also develop emerging models such as TME score available for identifying ICI responders.^15^, ^16^, ^17^ However, due to limitations in measuring these biomarkers, immune profiling of CRC still needs to be elucidated and multi‐dimensional biomarker discoveries are indispensable for precise ICI treatment.

Anti‐tumour immunity is not confined to the TME. Rather, a systemic immune response is also observed in patients. The broader tumour environment, known as the macroenvironment, consists of the tumour and surrounding organs responding to tumourigenesis. It has been shown that the cytotoxic T cells in TME are irreversibly exhausted and peripheric T cells are recruited to tumour sites elicited by immunotherapies, and therefore a systematic anti‐tumour immunity is essential for immunotherapeutic efficacy.^18^ Several studies also demonstrated immune perturbations systematically during tumourigenesis.^19^, ^20^, ^21^ Tumour macroenvironment including primary tumour, draining lymph node, bone marrow, spleen and blood has shown to undergo shift in the composition and function of immune cells in mouse models, suggesting that immune system was altered during tumour progression.^19^ Cachexia is another example of altered cancer macroenvironment by systemically metabolic reprogramming.^22^ On the other hand, immune cells could be resident in tumour environment and also have the ability to home by blood or lymphatic vessel.^23^ Tertiary lymphoid structures (TLSs) are atopic lymphoid tissues occurring in or surrounding tumour, suggesting a new connection between immune system and tumour in situ. However, so far tumour macroenvironment has been depicted mainly based on mouse models, which is not capable to accurately mimic human disease phenotypes, and we still lack a fully integrated view of immune landscape in CRC patients. Moreover, intestinal architecture is not homogeneous and makes up of epithelium, lamina propria, submucosa and muscularis propria where various immune cell types reside. Tumour cells originate from epithelium and propagate deeper into other layers of bowel wall, where TME may be heterogenous. And lymphocytic reaction to CRC has also been observed at the tumour invasive margins, which was particularly referred as Crohn's‐like reaction. Therefore, taking CRC immune macroenvironment into consideration helps understand immune profile of tumour subtypes and also contributes to diagnosis, precise treatment and prognosis prediction.^24^

Single cell and spatial technology provide unprecedented and detailed characterisation of heterogeneity of TME.^16^, ^25^ Single‐cell RNA sequencing (scRNA‐seq) has facilitated the characterisation of cell types at high resolution based on transcriptome despite limited cell number for detection. Cytometry by time‐of‐flight (CyTOF) is another single‐cell technique using heavy metal ion‐conjugated probes or antibodies, which provides expression of more than 40 markers in multiple samples with tens of thousands of cells at a time.^26^ Single‐cell technology has characterised the phenotypes of tumour cells, stromal cells and immune cells in the CRC TME at a high resolution.^16^ For example, the consensus molecular subtype (CMS), developed by bulk RNA‐seq, was also refined by scRNA‐seq, which demonstrated the intrinsic CMS (iCMS) of CRC. The iCMS2 and iCMS3 tumours could be well distinguished by differential expression gene (DEG) subsets recognised by scRNA‐seq data from large CRC cohorts.^27^ Furthermore, spatial omics including spatial transcription (ST) and spatial proteomics can map cell expression signatures to distinct geographical regions.^25^, ^28^ For example, interactions between FAP^+^ fibroblasts and SPP1^+^ macrophages were identified using both scRNA‐seq and ST, making it a potential therapeutic strategy to improve immunotherapy efficacy.^29^ However, there are limited studies charting CRC macroenvironment. Bulk RNA‐seq or scRNA‐seq of unstratified intestinal architecture is not able to decipher tumour immune macroenvironment. The shift in abundance and functional capacity of immune cells in the tumour macroenvironment, as well as the factors influencing immune macroenvironment, has not been unveiled. Couping large‐scale CyTOF with single‐cell and spatial whole transcriptomics sequencing illustrates systemic dysfunction and plasticity of immune cells based on cellular phenotype with detailed spatial context.

Overall, in the present study, we aim to characterise CRC immune macroenvironment to discover biomarkers for clinical utility of stratifying patients into ICI treatment. To this end, we develop a protocol of collecting spatial samples including tumour, peripheral blood mononuclear cell (PBMC), normal bowel and tumour–adjacent bowel tissues from CRC patients. A new anatomical dissection method is performed to acquire more detailed cell phenotype in each layer of gastrointestinal tract, ranging from epithelium to muscularis propria, and from ascending colon to rectum. Multiparameter CyTOF, scRNA‐seq and ST‐seq are exploited to elucidate the variation in the CRC immune macroenvironment and identify essential influencing factors, which correlate immune macroenvironment with patients’ response to ICI treatment. Thus, our findings could provide evidence and tools for adoption of immune macroenvironment in clinical decision.

## 2 METHODS

### 2.1 Patients and tissue samples

CRC patients who were treated‐naïve or received Toripalimab for 12 weeks and then underwent surgery were enrolled. Clinical information of patients was provided in Table S1.

Blood samples were obtained before surgery and PBMC was extracted by density gradient centrifugation using Ficoll–Paque. Tumour tissue, tumour margin, normal bowel tissue (5 cm away from tumour margin) and tumour–adjacent bowel tissue (less than 2 cm but more than .5 cm away from tumour margin) were obtained after surgery. Additionally, a colon adenoma was sampled and processed to scRNA‐seq. All sampled sites of tumour were confirmed by a surgeon, and stored in tissue storage solution (Miltenyi Biotec) at 4°C and immediately processed in laboratory.

### 2.2 Anatomical dissection of bowel

Four layers of human bowel were divided using a modified protocol from a previously report as described below.^30^ Muscularis propria was firstly separated by ophthalmic scissors and the remaining tissues were disposed in HBSS followed by dissecting mucosa and submucosa under stereomicroscope. Next, mucosa was cut into 5 × 5 mm, transferred to 10 mL epithelial cell solution (HBSS w/o Ca^2+^/Mg^2+^, 2% FBS, 10 mM EDTA, .5 mM DTT, 100 U/mL penicillin, 100 µg/mL streptomycin and 10 mM HEPES) and incubated for 25 min followed by vortexed for 15 s with another 5 min of incubation. Epithelial layer was separated from the underlying lamina propria by shaking vigorously, and then washed three times with Dulbecco's phosphate‐buffered saline (DPBS). Finally, the epithelial layer, lamina propria, submucosa and muscularis propria were collected in tubes for single‐cell suspension preparation.

### 2.3 Tissue dissociation and single‐cell suspension preparation

Tissues were minced to approximate 1 mm^3^, transferred to 10 mL enzymatic digestion mix (Base: RPMI1640, 2% FBS, 300 U/mL collagenase IV, 50 µg/mL Dnase I, 100 U/mL penicillin, 100 µg/mL streptomycin and 10 mM HEPES) and incubated at 37°C in a rotisserie rack with end‐over‐end rotation for 60 min. After digestion, all samples were quenched by 10 mM EDTA and the suspension was collected by passing through the 70 and 40 µm cell strainer. Cells were pelleted and red blood cells were lysed in ACK lysis buffer for 3 min on ice. After another wash and centrifugation at 300 × *g* for 5 min, cell pellet was resuspended in a solution of 90% FBS plus 10% DMSO and cryopreserved for CyTOF, when fresh cells were processed to scRNA‐seq.

### 2.4 Mass cytometry antibodies

All cytometry antibodies and concentrations used for test were listed in Table S2. Mental labelled antibodies were purchased from Standard BioTools. Conjugation of mass cytometry antibodies was prepared using either Maxpar antibody conjugation Kit (Standard BioTools) or Maxpar MCP9 antibody conjugation kit (Standard BioTools). Each antibody clone was titrated to optimal staining concentrations by testing on human PBMC. In order to confirm whether conjugated antibodies were suitable for Cell‐ID^Tm^ 20‐Plex Pd Barcoding Kit (Standard BioTools), all antibodies were tested in the preliminary experiment. The signal of each antibody clone was compared across three groups: staining without barcoding, staining before barcoding and staining after barcoding. A total of 40 antibodies were tested while five antibodies, including CCR4, CCR6, CCR7, CD138, CXCR3 were removed from the panel due to shifted signals after barcoding. Finally, a 35‐marker panel was applied for further experiment (Figure S1A and Table S2).

### 2.5 Cell preparation and measurement for mass cytometry

Cell preparation for mass cytometry was performed as previously described.^26^ Briefly, 1–3 × 10^6^ cells were thawed at 37°C and spun down. Cells were incubated in full culture medium at 37°C for 60 min, followed by washing and staining with Cisplatin (Standard BioTools) for 3 min. After another wash with Maxpar cell staining buffer (Standard BioTools), each sample was fixed in Maxpar Fix I Buffer (Standard BioTools) and barcoded with Pd isotopes. Cells were washed twice with cell staining buffer and pooled into a single tube for samples from the same patient. Pooled cells were then resuspended in cell staining buffer and incubated with True‐Stain Monocyte Blocker (Biolegend) to block Fc receptors for 10 min, followed by staining with surface antibodies cocktail for 30 min at room temperature. After staining, cells were washed twice by cell staining buffer, then fixed and washed twice by Maxpar Perm‐S buffer (Standard BioTools). Afterwards, cells were stained with intracellular antibodies cocktail for 30 min at room temperature. After two washes with cell staining buffer, samples were fixed by 1.6% formaldehyde (Thermo Fisher) prior to staining with 1:1000 iridium (Standard BioTools) in Maxpar Fix and Perm buffer (Standard BioTools) at 4°C overnight. And then, cells were washed once with cell staining buffer and twice with cell acquisition solution (Standard BioTools). Finally, cells were diluted in cell acquisition solution and added with bead standard (Standard BioTools) to appropriate concentration. Samples were processed using Helios (Standard BioTools) with approximately 300 events each second.

### 2.6 Single‐cell RNA sequencing

Single cells were processed through the DNBelab C4 Platform as per manufacture's recommendations. Briefly, single cells were partitioned into droplets generation, emulsion breakage and bead collection. Then cDNA was synthetised by reverse transcription and amplified to barcoded library. Next, cDNA was trimmed, linked with adaptor and amplified to generate sequencing library. Libraries were sequenced on a DNBSEQ‐T1 or DNBSEQ‐T7 sequencer at the China National GeneBank, ShenZhen, China.

### 2.7 Preparation and storage of samples for SpaTial Enhanced REsolution Omics‐sequencing (Stereo‐seq)

Surgical samples were rinsed by DPBS to remove red blood cells and immediately embedded in Optimal Cutting Temperature compound (OCT) followed by snap frozen in liquid nitrogen cooled isopentane. Frozen section was performed in cryotome for haematoxylin and eosin (H&E) staining and RNA extraction, and the tissue with RNA integrity number more than 7 was selected for Stereo‐seq.

### 2.8 Optimisation of permeabilisation time

Before generating the library of Stereo‐seq, the permeabilisation time of normal colon was optimised. Six minutes, 12 min, 18 min and 24 min were tested by mounting sequential four slice on fluorescent chips, followed by fixation, staining and then permeabilisation for different times. Fluorescent cDNA was then synthesised during reverse transcription and imaged by microscope. Finally, an 18‐min permeabilisation was performed for all samples.

### 2.9 Stereo‐seq library construction and sequencing

Stereo‐seq chip was washed twice in nuclease‐free water and then incubated with .1% poly‐L‐lysine for 30 min at room temperature. After being washed with water twice, the chip was dried at mental block at 37°C and immediately mounted with tissue section. Then the tissue section was dried at 37°C for 3 min and fixed in methanol at −20°C for 30 min. Following nucleic acid dye staining (Thermo Fisher) for 3 min, the chip was imaged using a Zeiss LSM880 confocal microscope. Subsequently, the tissue section was washed by .1 × Saline Sodium Citrate (SSC) with RNase inhibitor and permeabilised using .1% pepsin in .01 N HCl for 18 min. The mRNA released from permeabilised tissue was reversely transcribed at 42°C for 3 h. Next, tissue section was washed twice with .1 × SSC buffer followed by digestion using tissue removal buffer at 37°C for 10 min. The cDNA was released from the chip by incubated with Exonuclease I for no more than 18 h, and amplified by PCR with 15 cycles. Next, cDNA was purified by VAHTS DNA Clean Beads (Vazyme). Finally, cDNA was fragmented by Tn5 transposase at 55°C for 10 min, stopped by .02% SDS and mixed gently at 37°C. A 13‐cycle PCR was performed to acquire amplified cDNA library, which was purified by VAHTS DNA Clean Beads and then proceed to generate DNA nanoballs (DNB) for sequencing.

### 2.10 Bulk RNA‐sequencing

Approximately 1 µg of total RNA was prepared from frozen tumour samples for library construction. Oligo (dT)‐attached magnetic beads were used to purified mRNA which was fragmented into small pieces and then subjected to first‐strand cDNA synthesis using random hexamer‐primed reverse transcription, followed by a second‐strand cDNA synthesis. Subsequently, RNA index adapters were added after end repair and A‐tailing. The cDNA fragments were amplified by PCR. The double stranded PCR products from the previous step were heated, denatured and circularised by the splint oligo sequence to generate library. Finally, the library was amplified through rolling circle amplification to synthesise DNB and loaded to the patterned nanoarray, then sequenced on DNBSEQ platform with 100 bp paired‐end reads.

### 2.11 Multiplex immunohistochemistry (mIHC)

The TSA Opal multiplex immunostaining of tumour or bowel sections was performed. The paraffin‐embedded sections were treated with deparaffinisation, rehydration and epitope retrieval followed by blocking endogenous peroxidase activity with goat serum. Then, the sections were incubated with primary antibodies (SPP1, Thermo Fisher, #2417025, 1:200; CD68, Biolegend, #333802, 1:100; CD4, Abcam, #ab288724, 1:100; CD8, Santa, #A0516, 1:100; CD44, Abcam, #ab254530, 1:100; CD45, Abcam, #ab40763, 1:100; anti‐pan Cytokeratin, Abcam, #Ab7753, 1:100; α‐SMA, Abcam, #Ab5694, 1:100; ISG15, Abcam, #ab285367, 1:100) according to panel sequentially overnight at 4°C. Secondary antibody and fluorophore were applied per round. Sections were then counterstained with 4',6‐Diamidino‐2‐phenylindole dihydrochloride (DAPI, Thermo Fisher) and mounted using ProLong Gold Antifade mounting media (Invitrogen). Finally, the slides were scanned and analysed by TissueFAXS and StrataQuest (TissueGnostics).

### 2.12 Masson's trichrome staining

The tissue samples from tumour patients were immersed in 4% paraformaldehyde and embedded in paraffin. Then samples were stained using Masson trichrome staining kit to evaluate the degree of stroma. Tumour stroma is determined by the ratio of collagen fibres to the total surface area of the collagen fibres. The fibrotic area and total detected area in slice were assessed using ImageJ2.

### 2.13 Measuring the density and size of TLS in CRC patients

At least three stereoscopic views of H&E staining slices including tumour, invasive margin and adjacent bowel were scanned and analysed by QuPath (V0.5.0) software. The number of TLS was counted when total area of slices was also measured (Tables S1 and S8).

### 2.14 Concatenation, debarcoding and normalisation of mass cytometry data

After data acquisition, mass cytometry data were normalised according to the signal of batch intercalated bead standard, which provided intensity values of a sliding window to correct instrument fluctuations in channels over time and between samples.^31^ After normalisation, files were concatenated and debarcoded, using the debarcoding algorithm in software CyTOF.

### 2.15 Gating, compensation and batch effect removal of mass cytometry data

All mass cytometry files were input to FlowJo™. After gating out beads, singlets were identified by gating based on event length and iridium, followed by selection of live cells at cisplatin‐negative cells and CD45^+^ immune cells at CD45 positive cells. Sequentially, single and live CD45^+^ immune cells were output to be analysed by R. Expression values were transformed using an inverse hyperbolic sine (arcsinh) transform with cofactor 5. The compensation for channel signals was performed using the compensation matrix from R package CATALYST as previously reported.^32^ To mitigate the batch effect, two algorithms of batch effect removal, CytoNorm and cyCombine, were tested on our CyTOF data. Earth Mover's Distance (EMD) reduction sore was utilised to evaluate the effect of batch effect removal.^33^, ^34^ Batch‐corrected data were only used for principal component analysis (PCA), Uniform Manifold Approximation and Projection (UMAP) and clustering, when original expression data were retained for marker differential expression analysis, which was also reported in the analysis pipeline of scRNA‐seq.^35^

### 2.16 Clustering of mass cytometry data

We used FlowSOM for clustering, which was based on Self‐Organising Map and worked quickly and well in large CyTOF data.^36^, ^37^ A total of 29 major clusters were identified after meta‐clustering based on the expression of 30 lineage markers, except five functional markers including CD103, CD69, PD‐1, CD39 and Ki‐67. To validate the accuracy of cluster annotation, we calculated marker enrichment modelling (MEM) score for each marker of each cluster.^38^

### 2.17 Processing CO‐Detection by indEXing (CODEX) data

The colon data available on the HuBMAP Portal were either download or processed according to the pipeline of Garry P. Nolan Laboratory.^39^ The raw experiment folder data acquired from microscope were processed using CODEXUploader to tile files containing all cycle, channels and z‐slices. Segmentation was performed by using CODEXSegm with following parameters: radius = 6, max cutoff = .99, min cutoff = .05, relative cutoff = .2, cell size cut off factor = 1. The compensated expression matrix along with the cell coordinates were analysed by R package Seurat. Briefly, centred log‐ratio‐based normalisation was applied to protein data followed by dimensional reduction and graph‐based clustering. Manual annotation of mucosa, submucosa and muscularis propria in intestinal segments was done by R package SPATA. To map immune cells to regions of epithelial layer, lamina propria, submucosa and muscularis propria, we calculated the distance of all cells to epithelial cells. Intra‐epithelial immune cells were defined as cells located within one‐cell distance away from epithelial cells.

### 2.18 Processing raw data of scRNA‐seq

R package Seurat was used to analyse scRNA‐seq data. All cells of samples were combined and filtered based on the following criteria: cells with >250 genes, >1000 unique molecular identifier (UMI) and <20% mitochondrial gene expression in UMI counts were selected for further analysis. Ambient RNA was reduced using SoupX with default parameters. The rho value estimated by autoEstCont function was all below .05.^40^ Then doublets were removed using Scrublet when the threshold of doublet score was set to .22.^41^

The gene expression matrices were normalised by the total UMI counts with each cell scaled to the pseudocount 10 000 followed by a log‐transformation. A total of 2000 most variant genes were identified by FindVariableFeatures function and selected for PCA. ElbowPlot function plotted the standard deviations of the principal components and determined the number of principal component (PC) using for UMAP and constructing a K‐nearest neighbour graph. FindNeighbors function used K‐nearest neighbour graph to construct a Shared Nearest Neighbour (SNN) graph by calculating the neighbourhood overlap and applied Approximate Nearest Neighbours Oh Yeah for nearest neighbour finding. Then we used FindCluster function which applied Louvain algorithm to identify clusters, and resolutions from .2 to 1.2 were explored for the best clustering. Little batch effect was observed. We compared expression of each sub‐clusters to others by FindAllMarkers function using a Wilcoxon rank‐sum test with Bonferroni–Hochberg procedure.

### 2.19 Ligand–receptor expression and cell interactions

Cellchat was applied to evaluate cell–cell interactions.^42^ Clusters with less than 10 cells were removed before analysis. Cell status‐specific communications were inferred based on overexpressed ligands or receptors. To overcome the shallow sequencing depth of scRNA‐seq data, gene expression data were also projected to the protein‐protein interaction network. Next, cell–cell communication network was inferred by assigning each interaction with a probability value and performing a permutation test. The effect of cell proportion in each cell group in the probability calculation was also considered. Intercellular communication network of each sample was then quantified, visualised and compared.

### 2.20 Processing Stereo‐seq fastq reads into gene expression matrices

The first‐run Fastq files were generated to acquire cell identity (CID) messages, when the second run of Fastq files consisted of cDNA sequence, with 1–25 bp of read 1 for CID, 26–35 bp for molecular identity (MID) and read 2 for cDNA. CID sequences were mapped to the first‐round files to recover coordinates, allowing tolerance for 1 base mismatch due to sequencing and PCR error, while 2 bases with quality lower than 10 in MID was tolerant. For cDNA sequence, STAR was used to map sequencing data to human reference genome (GRCh38). Finally, mRNA reads and their coordinates were used to generate a CID containing expression profile matrix.

### 2.21 Image registration and calling gene expression under the area of tissue

After imaging tissue nuclei with microscope, ImageJ was used to stitch tiles. Images was then registered to the virtual chip image generated based on coordinates of gene expression using MATALAB. Then the registered image was binarised and noise was removed by two‐dimensional (2D) median filtering. The gene expression matrix was binned to 100 × 100 bins. One bin represents one DNB, with a radius of 220 nm, and the distance from centre to centre is 500 nm. The binned expression matrix was subsequently masked by binarised image matrix. The size of registered image was reduced to 2000 pixels in its largest dimension and the scale factor of image was recorded. The processed gene expression matrix, matched coordinates, registered and scaled image and scale factor were all required for the following analysis.

### 2.22 Processing Stereo‐seq data

Bins with >500 genes and <30% mitochondrial gene expression in UMI counts were selected for further analysis. The gene expression matrices were normalised by R package Seurat SCTransform which used regularised negative binomial regression for modelling single‐cell expression data and also performed well in ST data. Genes which are highly variable across the spots were detected using the Pearson residuals computed in the normalisation step, and 3000 most variant genes were selected for PCA. Following plot of the standard deviations of the PCs, the first 15 PCs were utilised to perform UMAP and SNN‐based clustering. Differentially expressed genes were identified by performing fold change analysis with Wilcoxon rank‐sum test. *p* values were adjusted by Bonferroni–Hochberg procedure.

### 2.23 Integrating spatial transcription and scRNA‐seq data

To estimate the cell abundance in Stereo‐seq slides, we combined reference single‐cell transcriptome and spatial transcriptome to run cell2location algorithm.^43^ Briefly, we defined two hyperparameters: (1) the expected cell abundance was set to be 10 cells each spot, determined by counting average number nuclei in each 100 × 100 bin at the registered images; (2) the regularisation strength of detection efficiency effect was set to be 200. The number of training iteration was 30 000. The posterior distribution of cell abundance estimated by cell2location was used for downstream visualisation and co‐localisation analysis. Additionally, we also applied anchor‐based integration workflow in Seurat with default parameters.

### 2.24 Correlation of expression of ligand/receptor and the abundance of corresponding cell type in spatial transcription

The correction between gene expression level of pair ligand/receptor and the abundance of corresponding cell type in each spot was demonstrated by Pearson correlation of gene expression of ligand/receptor and cell abundance in that spot and its surrounding spots. For Visium data, the number of surrounding spots was 6. First dimension value of PCA of the gene expression of ligand/receptor and cell abundance of targeted cell type were used to calculate Pearson correlation coefficient.

### 2.25 CMS classification for bulk RNA‐seq and ST‐seq data

DeepCC,^44^ CMScaller,^45^ CMSSSP and CMSclassifier^46^ algorithm were utilised to identify CMS of CRC based on RNA‐seq data. Tumours were assigned only the CMS phenotype that was consistent across the results of at least two algorithms; if the imputation results were contradictory, they were labelled as ‘non‐consensus’. For classification of iCMS, we calculated the Gene Set Variation Analysis (GSVA) score of iCMS2 and iCMS3 metagene expression scores using the 715 iCMS marker genes as previous reported.^27^ And hierarchical clustering was then performed to subclassified tumours into iCMS2, iCM3 or non‐consensus group.

### 2.26 Statistical analyses

R (version 4.1.3) was used for statistical analyses and graphing. To identify the shift of cell abundance, the frequency of cell population was used for analysis by R package diffcyt, which applied negative binomial generalised linear model from R package edgeR and performed likelihood ratio test for differential abundance. *p* values were adjusted using the Benjamini–Hochberg procedure to control false discovery rate. For differential status testing, two‐sided Student's *t*‐test was used to determine statistical significance between two groups, and *p* values were adjusted using the Benjamini–Hochberg procedure if multiplexed test was performed. The one‐way analysis of variance (ANOVA) was performed to determine whether there were statistically significant differences among more than two groups, when Tukey's post hoc test was used for multiple comparisons.

## 3 RESULT

### 3.1 The overview of the single cell and spatial landscape of the immune macroenvironment in CRC

**FIGURE 1:**
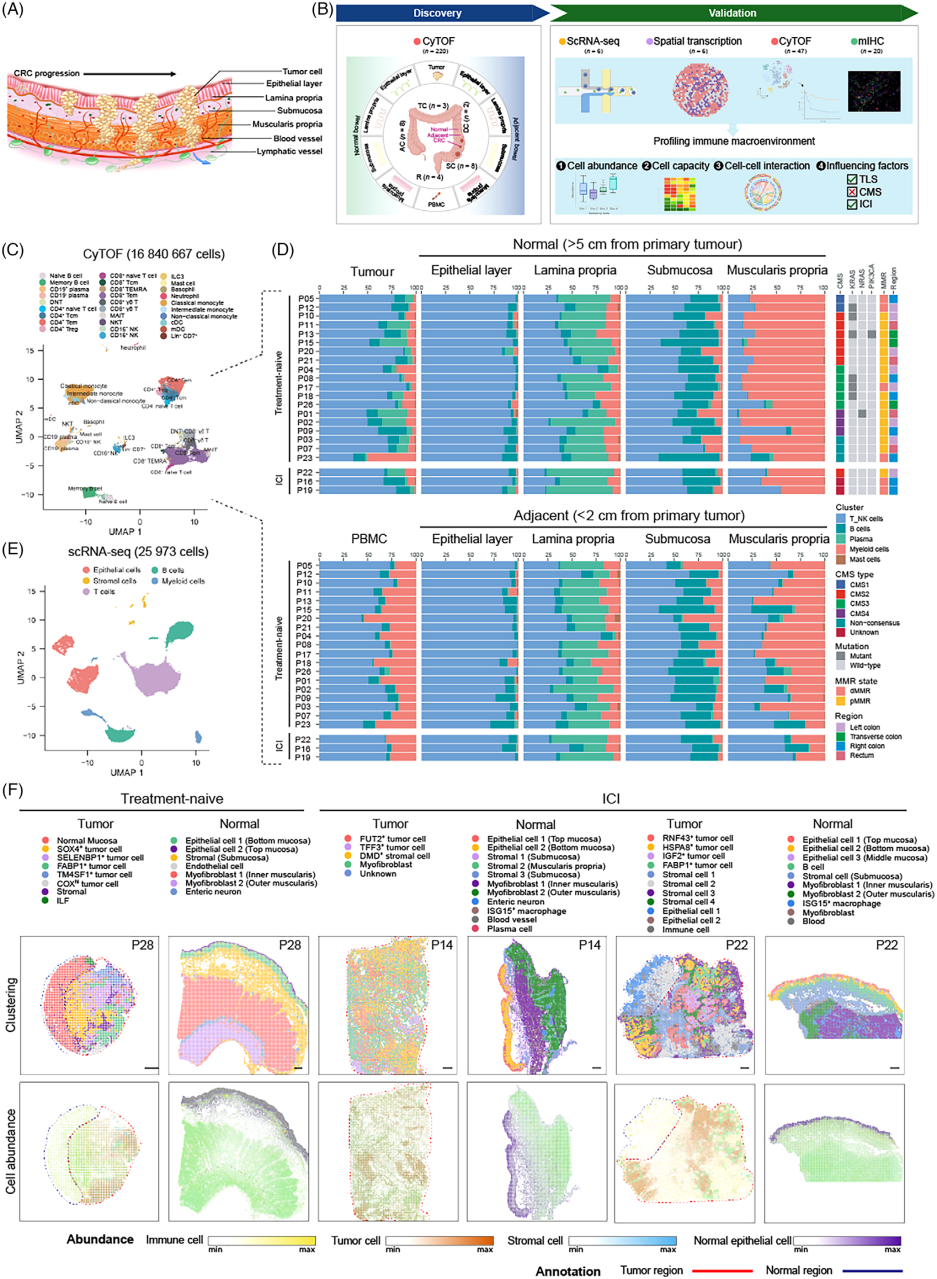
Single cell and spatial landscape of the colorectal cancer (CRC) macroenvironment. (A) Graphic overview of CRC progression and the anatomy of human bowel wall. (B) Experimental strategy and analytical pipeline. (C) Uniform Manifold Approximation and Projection (UMAP) representation of analysed cell populations based on the expression of 30 lineage markers in cytometry by time‐of‐flight (CyTOF) data. (D) Bar plots illustrating the proportions of the major five cell types in 10 different locations from 22 CRC patients. The information of consensus molecular subtype (CMS), mutation status, mismatch repair status and regions of tumour were aligned at right side. (E) The UMAP plot showing the major five cell types identified in single‐cell RNA sequencing (scRNA‐seq) data. (F) Representative images showing the spot clustering (upper) and histopathological regions annotated (lower). Scale bars = 500 µm. ICI, immune checkpoint inhibitor; TLS, tertiary lymphoid structures.

To elucidate the composition and functional capacity of immune cells from various tissue structures in the CRC macroenvironment as well as influencing factors (Figure 1A), samples including PBMC, tumour, normal bowel and tumour–adjacent bowel tissues were enrolled in this study, and CyTOF, scRNA‐seq and ST‐seq were applied to created multi‐omics data for discovery and validation (Figure 1B, Methods). To begin with, we applied a modified method to dissect human bowel into four layers including epithelial layer, lamina propria, submucosa and muscularis propria.^30^ These layers were confirmed to be well separated by H&E staining and immune fluorescent staining results (Figure S1B–G). A 35‐marker CyTOF panel, which covered CD45^+^ immune cell types, was used to quantify immune profile (Figure S1A and Table S2). As a result, a total of 220 spatial samples from 19 treatment‐naïve patients and three patients with anti‐PD‐1 therapy were processed for CyTOF, followed by the analysis pipeline including gating, data transformation, removal of batch effect and clustering (Figure S1H–N, Methods). In addition, we also acquired 20 samples from another four patients including bulk digested bowel samples and culture tumour‐infiltrating lymphocytes (TILs), to cover all lineages and evaluate the differences in cell type abundance between our method of tissue disassociation and the traditional method (Figure S1O). Then CyTOF data of bulk digested bowel samples and culture TILs were removed for further analysis. Finally, a total of 16 840 667 cells were recovered, with 29 clusters identified, ranging from 1411 to 363 267 cells for spatial samples in the discover cohort (Figures 1C and S1H). We obtained five broad cell types in spatial samples including T/NK/ILC cells, B cells, plasma cells, myeloid cells and mast cells, indicating apparent immune profiles across different sites (Figure 1D).

Next, whole transcriptome sequencing including RNA‐seq, scRNA‐seq and ST‐seq was performed. We generated scRNA‐seq profiles of cells isolated from epithelial layer, lamina propria, submucosa and muscularis propria of normal colon, tumour tissue, as well as adenoma tissue from one patient in validation cohort. A total of 25 973 transcriptomes were obtained after quality control (Figures 1E and S2A). Clustering analysis split these into five compartments. Furthermore, to map spatial distributions of transcriptomes, we undertook ST on tumour and normal bowel tissues from three patients with or without ICI treatment. After quality control of spots, unsupervised clustering analysis identified 5–11 clusters in each slide, when spatial mapping of cell types showed the overall organisation of epithelial cells, immune cells and stromal cells in each tumour and normal bowel (Figures 1F and S2B). The layers of three normal bowel samples demonstrated consistent expression patterns (Figure S3B,D,F). For example, spots under the mucosa showed strong expression of epithelial markers such as PIGR, ZG16, FABP1, GUCA2A and CEACAM7, as well as plasma cell markers including IGHA1, IGHA2 and IGKC. In the submucosa, genes indicative of stromal cell, such as COL1A1, DCN, VIM, LUM, NDE1 and CDH4, were highly expressed. Interestingly, the muscularis propria could be divided into two distinct layers: the inner ring contained circular muscle fibres, expressing higher MYL9 and MYH11, while outer ring was composed of long muscle fibres, overexpressing FOS, JUN and JUNB, which were reported to be expressed in skeletal muscle cells.^47^ This differential gene expression pattern of muscularis propria may indicate different function of muscle fibres. Nerve fibres were also found in submucosa and areas between circular and long muscle fibres. On the other hand, tumour cells were heterogeneous across tumour slices from different patients, as indicated by largely expressed makers in tumour regions, when stromal cells and immune cells were also identified (Figure S3A,C,E). Taken together, we generated a single cell and spatial landscape of CRC immune macroenvironment.

### 3.2 Immune profiling of human bowel identified distinct immune cell niches

**FIGURE 2:**
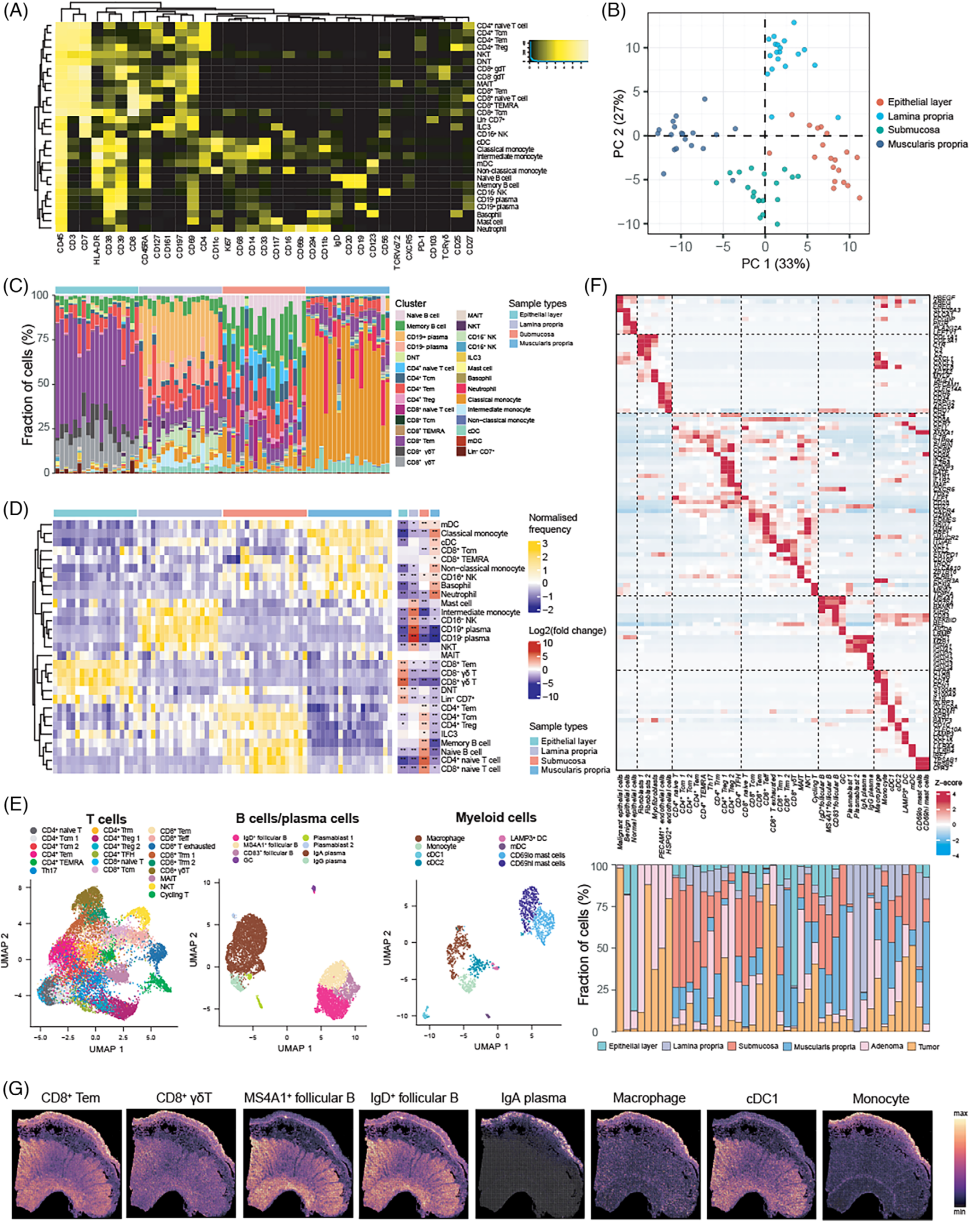
Immune profiling of the human bowel revealed by multi‐omics. (A) Heat map indicating the normalised expression of 35 markers in 29 immune cell clusters identified by cytometry by time‐of‐flight (CyTOF). (B) Principal component analysis (PCA) of cell type composition. (C) The cell subset proportions across normal bowel samples identified by CyTOF. (D) Heat map of each cell cluster frequency, by row, in each site. Arcsine transform was performed for frequency. (Left) Log2 fold‐change of proportion and significance were aligned on the right side (*p < .05; **p < .01, likelihood ratio test; *p* values were adjusted by Benjamini–Hochberg procedure). (E) Uniform Manifold Approximation and Projection (UMAP) representation of immune cell populations based on single‐cell RNA sequencing (scRNA‐seq) data. Cells were coloured by cell types. (F) Heat map of scaled mRNA expression of representative markers defining each cluster in scRNA‐seq data (upper). Cell subset proportions were shown by bar plot (lower). (G) Deconvoluted immune cell abundance in a normal bowel sample by cell2location algorithm.

To begin with, our CyTOF data quantified the composition and activity status of immune cells in the spatial context of human bowel, taking sites that encompassed the epithelial layer, lamina propria, submucosa and muscularis propria, as well as segments ranging from the ascending colon to rectum into consideration. These data provided a baseline of immune profiles for analyses of tumour perturbation. Based on the expression of proteins, a total of 29 immune cell subsets were identified including CD4^+^ T cells, CD8^+^ T cells, double negative T cells (DNT), mucosal‐associated invariant T cells (MAIT), monocytes, dendritic cells (DC) including myeloid DC (mDC) and conventional DC (cDC), B cells, and plasma cells (Figure 2A). The accuracy of clustering was further confirmed by enrichment scores (Figure S4A and Table S3). PCA based on cell abundance demonstrated the distinct profile of four layers (Figure 2B). The frequency of cell types revealed four significant compartments (Figure 2C,D). As for epithelial layer, CD8^+^ Tem cells, CD8^+^ γδ T cells, CD8^−^ γδ T cells, DNT and innate immune cells (Lin^−^ CD7^+^) were enriched, while mast cell, intermediate monocyte, CD16^−^ NK cells, CD19^+^ or CD19^−^ plasma cells and NKT cells were specifically enriched in the lamina propria. On the other hand, most of CD4^+^ T cell subsets such as CD4^+^ naïve T cell, Tem, Tcm, Treg, B cell subsets such as naïve B cell as well as memory B cell, and group 3 innate lymphoid cells (ILC3) were the major immune cell types in the submucosa. As for muscularis propria, myeloid cell subsets such as classical and non‐classical monocyte, mDC, cDC, neutrophil and basophil, as well as CD16^+^ NK cells, CD8^+^ Tcm and CD8^+^ T_EMRA_ were predominant cell types.

We also inquired the transcriptomic features of immune cells in the CRC macroenvironment. Graph‐based clustering was performed to identify epithelial, stromal and immune cells, followed by sub‐clustering immune cells into subsets of T cells, B cells and myeloid cells (Figures 2E and S4B). A total of 45 subsets were identified including 3 epithelial subsets (comprising one malignant epithelial cluster, one benign epithelial cluster and one normal epithelial cluster), five stromal cell subsets, 21 T cell subsets (including 10 CD4^+^ T cell subsets, eight CD8^+^ T cell subsets, one MAIT subset, one NKT subset, one cycling T cell subset), eight B cell subsets and eight myeloid subsets. Each cell type was supported by representative markers (Figure 2F). As for immune cells, scRNA‐seq revealed that the cell abundance of immune cell types from different layers in normal colon was in line with the results of CyTOF data. This indicated the enrichment of CD8^+^ γδ T cells in the epithelial layer, plasma cells and mast cells in the lamina propria. There was also an increase in infiltration of T cell subsets including CD4^+^ naïve T cells, CD4^+^ Tcm cells, Treg, CD8^+^ naïve T cells and follicular B cell subsets in the submucosa, as well as enrichment of monocytes, cDCs in the muscularis propria (Figure 2F).

Next, we integrated our single cell and ST data to unveil the distribution of immune cells in spatial context (Figure 2G). The spatial abundance of each cluster was similar to that observed in CyTOF and scRNA‐seq. For example, CD8^+^ Tem and CD8^+^ γδ T cells were mostly enriched at the apical epithelial layer. Follicular B cells were aggregated at isolated lymphoid follicle (ILF) within mucosa but exhibited a relatively low abundance in the whole slide. Plasma cells expressing IgA were specifically enriched in the mucosa. As for myeloid cells, macrophages and monocytes were highly enriched in the mucosa, although they were also distributed in other layers. Since there was no apparent ILF structure within the submucosa of our colon slice, we additionally utilised another ST dataset of colon slices containing ILF for analysis. As a result, germinal centre (GC) B cells and naïve B cells were enriched in ILF which was located at submucosa (Figure S4C). Moreover, mIHC staining of human colon form Human BioMolecular Atlas Program (HuBMAP)^48^ also showed a similar organisation of immune cells (Figure S4D–G).

We have revealed distinct immune profiles in each layer of bowel. However, the compositions of immune cell type which were located at different segments of bowel ranging from ascending colon to rectum should be also taken into consideration. As shown, the composition of immune cell types seemed harmony across colon and rectum, with the exception of decreased CD8^+^ naïve T cells and increased classical monocytes in the submucosa of the rectum compared to the right colon, as well as fewer neutrophils in muscularis propria of the rectum compared to the right colon (Figure S4H,I). Altogether, our study provided a deeper understanding of spatial distribution of immune cells in human bowel, serving as a baseline of immune profiles for studying altered cell composition and functional capacity under disease conditions.

### 3.3 Immune profiling of tumour–adjacent bowel and tumour described an immunosuppressive environment

**FIGURE 3:**
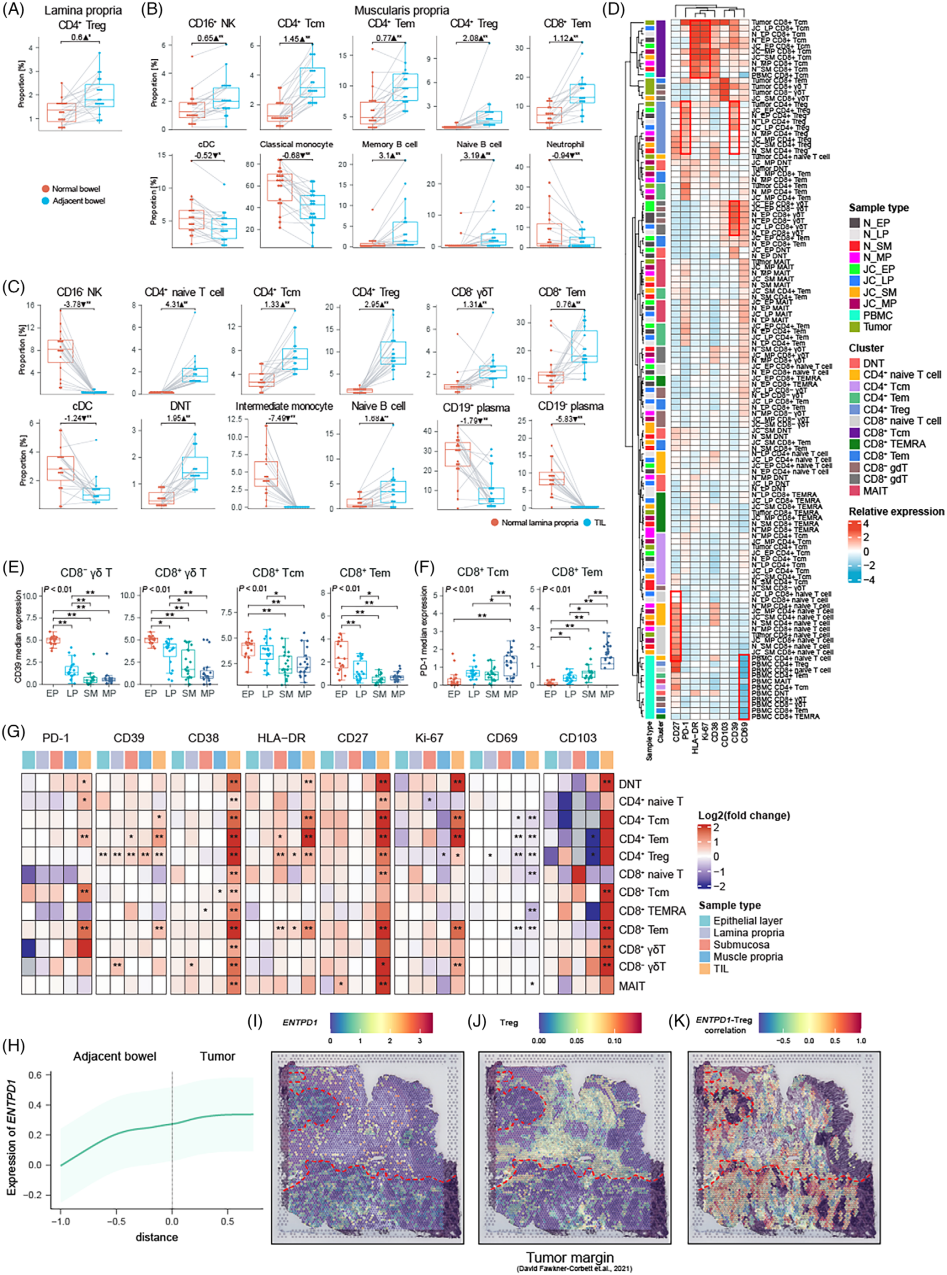
Characterising an immune‐suppressive environment surrounding colorectal cancer (CRC). (A, B) Comparison of the proportion of indicated CD45^+^ clusters in the lamina propria and muscularis propria between normal bowel and juxta‐cancerous bowel tissues. (*n* = 19 samples per condition. Likelihood ratio test was performed. *p* values were adjusted by Benjamini–Hochberg procedure.) (C) Comparison of the proportion of CD45^+^ clusters between tumour tissue and normal lamina propria. (*n* = 19 samples per condition. Likelihood ratio test was performed. *p* values were adjusted by Benjamini–Hochberg procedure.) (D) Heat map showing the scaled protein expression of T cell subsets in each site. (E, F) Box plots depicting the normalised expression of CD39 (E) or PD‐1 (F) in different T cell subsets. All groups were analysed by one‐way analysis of variance (ANOVA) tests and Tukey's post hoc tests. (G) Comparison of the protein expression of each T cell subset in each bowel layer between normal bowel and adjacent bowel, with comparison to the counterparts in the normal lamina propria. *T*‐test was performed and *p* values were adjusted by Benjamini–Hochberg procedure. (H) Predicted mRNA expression of ENTPD1 over distance measured from the tumour margin. The data were shown as mean ± standard deviation. (I) Normalised expression of ENTPD1 in spots covering the margin of CRC. Tumour margin was indicated by red lines. (J, K) Spatial plots of deconvoluted Treg abundance (J) as well as the correction between Treg abundance and normalised expression of ENTPD1 (K). In all box plots in this article, the horizontal line in the middle of each box corresponds to the median when the top and bottom borders of the box delineate the 75th and 25th percentiles, respectively. The top or bottom whisker extends from the hinge to the largest or smallest value, no further than 1.5× the inter‐quartile range, respectively. Number above bracket, log2 fold‐change; ▲increased; ▼decreased; *p < .05; **p < .01. EP, epithelial layer; JC, juxta‐cancerous; LP, lamina propria; MP, muscularis propria; N, normal; SM, submucosa; TIL, tumour infiltrated lymphocyte.

Based on the immune landscape of normal bowel tissues, we compared the component of immune cell in each bowel layer between tumour–adjacent sites and normal sites. As for tumour–adjacent bowel, CD4^+^ Treg cells were increased in lamina propria, when several T cell and B cell subtypes were increased in muscularis propria, such as CD4^+^ Tcm, CD4^+^ Tem, CD4^+^ Treg, CD8^+^ Tem, naïve B cell and memory B cells (Figure 3A,B). In contrast, myeloid cell subsets were decreased in muscularis propria, such as cDC and classical monocytes. Notably, there were no significantly altered immune cell types observed in epithelial layer and submucosa during tumourigenesis.

The different abundance of infiltrated immune cells between primary tumour and normal tissue revealed the dominant immune cells in TME. As shown in Figure 3C, subsets of T cells and B cells were remarkably increased, when myeloid cell subsets and plasma cells were dramatically decreased in TME compared to their counterparts in the normal lamina propria.

Next, we investigated the expression of functional capacity of T cells in the macroenvironment (Figure 3D). Interestingly, hierarchical clustering showed the same cellular populations in different sites were likely to be clustered together, despite PBMC, mainly due to the low expression of CD69, a marker of tissue‐resident T cells. There were markers up‐regulated in specific T cell subsets, such as CD27 in naïve T cells, PD‐1 and CD39 in Treg, HLA‐DR and Ki‐67 in Tcm cells, as well as CD39 in γδ T cells. Moreover, variable expression of markers across different sites was also observed, like increasing expression of CD103 in CD8^+^ T cells from tumour tissue. We further described the shift of T cell function. First of all, we compared the expression level of functional markers in immune cells across different layers of the normal bowel. We found that CD39 was overexpressed in γδ T cells, CD8^+^ Tcm cells, CD8^+^ Tem cells, with a decreasing trend from the epithelial layer to the muscularis propria (Figure 3E). On the other hand, the expression of PD‐1 in CD8^+^ Tcm and Tem showed an increasing trend from the top to the bottom layers of the bowel (Figure 3F). These results indicated that CD8^+^ T cells situated in different layer of bowel wall have various functional capacities. Next, we compared the protein expression of each T cell subset in each bowel layer between normal bowel and adjacent bowel, as well as tumour tissue, revealing an altered spatial pattern of gene expression under the perturbation of tumour (Figure 3G). The expression of PD‐1 was increased in DNT, CD4^+^ naive T cell, CD4^+^ Tem, CD8^+^ Tcm and CD8^+^ Tem cells in the tumour site, while the expression of CD39 was also up‐regulated in CD4^+^ Tcm, CD4^+^ Tem, CD4^+^ Treg and CD8^+^ Tem cells. Moreover, the expression level of CD39 was altered in several T cell subtypes from tumour–adjacent bowel, such as CD4^+^ Tem, CD8^+^ Tem, CD8^−^ γδ T cells and especially CD4^+^ Treg, indicating that CD4^+^ Treg promoted the immune‐suppressive environment during tumour progression at the margin of tumour. As for activation markers, the expression of CD38 was increased in all T cell subtypes in the tumour sample, while HLA‐DR and CD27 were also up‐regulated in numerous T cell subsets. CD69, a marker of recently activated or tissue‐resident T cells, was decreased in several T cell types in the tumour and adjacent bowel layers. The integrin CD103, responsible for cross‐talk between T cells and epithelial cells, was up‐regulated in tumour‐infiltrating T cells. CD103^+^ CD39^+^ CD8^+^ TILs were recognised as tumour‐specific T cells, and it was reported that these cells also expressed low levels of CD69.^49^, ^50^, ^51^ Therefore T cells in the TME and tumour–adjacent bowel showed tumour‐specific phenotype. To further estimate the spatial context of perturbed T cells in the surrounding area of tumour, we mapped cells to a slice of tumour margin by integrating our scRNA‐seq data and published ST data. As a result, we found that the expression of CD39 tended to be increased near the tumour, with spots under stromal region displaying higher expression of CD39 and an increasing infiltration of Tregs (Figure 3H–J). The correlation between the expression of CD39 and the abundance of Tregs in ST data indicated that Tregs contributed to the expression of CD39, and the correlation was not observed in other cell types, which confirmed the shift in the expression of CD39 in Tregs as identified by CyTOF (Figure 3K). In general, our results revealed the terminally activated and exhausted status of T cells in the tumour, when CD39^+^ CD4^+^ Tregs were increased in the marginal intestinal architecture, potentially contributing to the establishment of immunosuppressive environment and tumour progression.

### 3.4 Global intercellular communications unveiled the immunosuppressive role of the SPP1–CD44 interaction in the CRC macroenvironment

**FIGURE 4:**
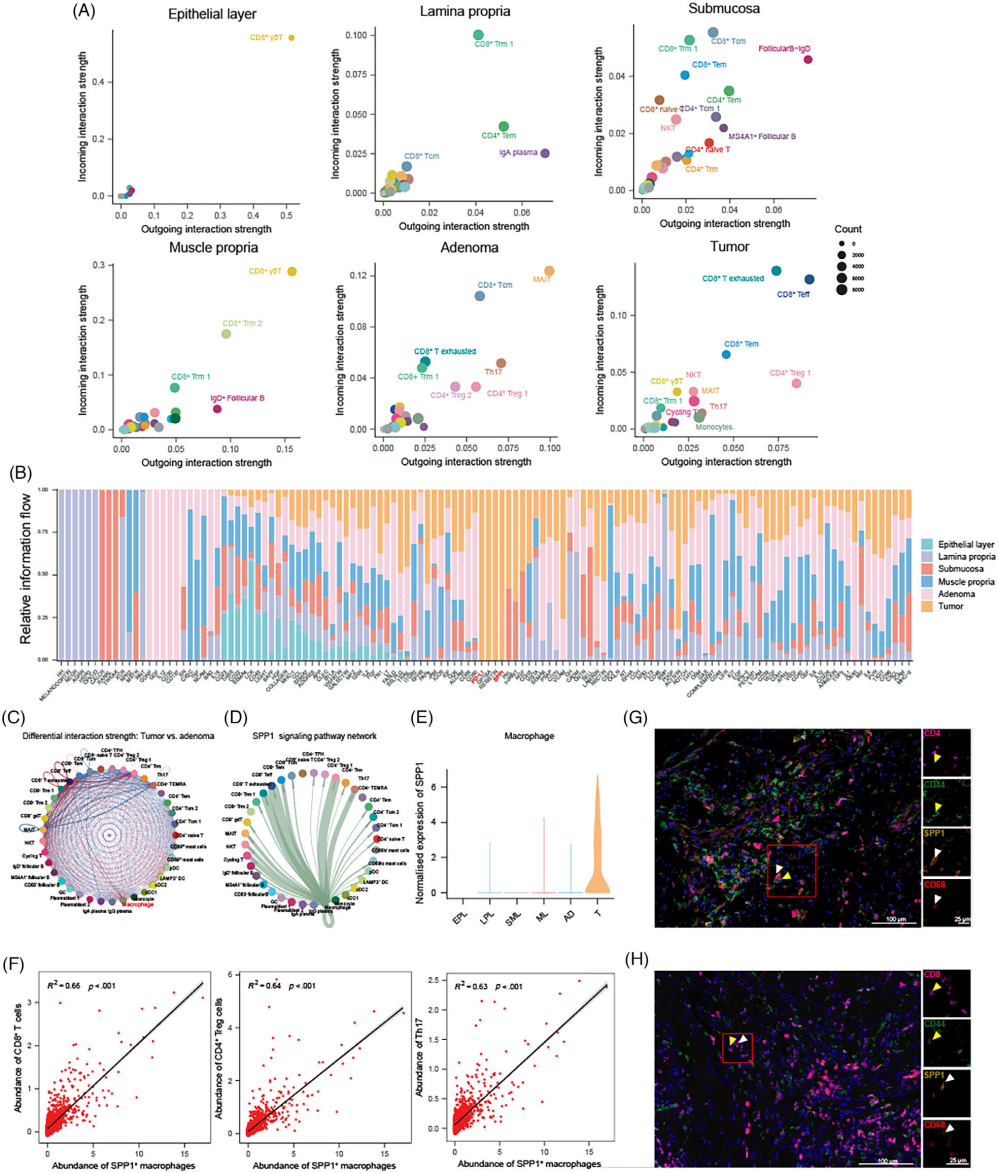
Cell–cell communications in the colorectal cancer (CRC) macroenvironment indicated immune‐suppressive function of the SPP1–CD44 interaction. (A) The interaction strength of immune cells in different sites. Dot size represents counts of interaction. (B) Bar plot showing the proportion of interaction pathways across samples identified by single‐cell RNA sequencing (scRNA‐seq). (C) Comparison of interaction strength of each immune cell type between adenoma and tumour. (D) The interaction of immune cell types in the tumour through SPP1 signalling pathway. (E) The expression of SPP1 in macrophages from epithelial layer, lamina propria, submucosa, muscularis propria, adenoma and tumour. (F) Scatter plot showing the correlation between the abundance of SPP1^+^ macrophages and abundance of CD8^+^ T cells, CD4^+^ Treg cells, as well as Th17 cells. The error band indicates 95% confidence interval. (G, H) Immunofluorescence staining of SPP1^+^ CD68^+^ macrophages and CD44^+^ CD4^+^ cells (G) or CD44^+^ CD8^+^ cells (H) in CRC tissues. White arrows indicated SPP1^+^ CD68^+^ macrophages, and yellow arrows indicated CD44^+^ CD4^+^ cells or CD8^+^ cells.

We have unveiled disturbed immune cell compositions and increasing immunosuppressive marker expression of T cells in the CRC macroenvironment. Next, we analysed altered intercellular communications under the condition of tumourigenesis and aimed to interpreted the dominated immunosuppressive regulation based on our scRNA‐seq datasets of tumour, adenoma and stratified normal bowel samples. The number of interactions in immune cells from epithelial layer was relatively low, with interactions between T and B cell subsets being the only one detected (Figure S5A). Besides, CD4^+^ T cells were involved in the most interactions in all samples, except for the epithelial layer. More interactions between plasma cells and CD4^+^ T cells were observed in the lamina propria, and interactions between DC and T cells were increased in the submucosa. We also estimated dominant sources and targets in the communication networks, highlighting the important roles of specific cell types in cell–cell communications (Figure 4A). As shown, the cell types with most interactions were almost overlapped with enriched cell types in each site. For example, CD8^+^ γδ T cells in epithelial layer showed the highest interaction strength, the proportion of which was also increased in the epithelial layer. However, there were also specific cell types contributing to strong interaction strength which were not enriched in abundance, such as CD8^+^ Trm1 in lamina propria and CD8^+^ γδ T cell and CD8^+^ Trm2 in the muscularis propria.

Next, we summarised the interaction landscape of signalling pathways at each site (Figure 4B). Several interactions only occurred in specific locations, such as signalling pathways of CALCR, RANKL and TWEAK in the submucosa, GDNF, GDF, IL12, CD30 and CD137 signalling pathways in the adenoma, and PD‐L1, GP1BA, RESISTIN and SPP1 signalling pathways in tumour. The enriched ligands or receptors at each site were highlighted at Figure S5B.

To recognise rewiring interactions in TME, we identified the different cell–cell interactions between tumour and adenoma, revealing up‐regulation of interactions between macrophages and CD8^+^ T cells in tumour (Figure 4C). Macrophages interacted with nearly all immune cell types specifically exhausted CD8^+^ T cells (CD8^+^ Tex) CD8^+^ Teff, CD4^+^ Treg 1 and Th17 by SPP1–CD44 interaction (Figure 4D), which only occurred in tumour sites, and no expression of SPP1 in macrophages from normal bowel tissue and adenoma was observed (Figure 4E). Next, we investigated SPP1–CD44 interaction in situ. As shown by integrating scRNA‐seq and ST‐seq data, macrophages were enriched in spots surrounding tumour cells (Figure S5C), validated by the expression of SPP1 (Figure S5D). CD44 was highly expressed in nearly all regions (Figure S5E). We further investigated the correlation between macrophage abundance and the strength of SPP1–CD44 interaction in each spot. Only regions with enriched macrophages demonstrated a high Pearson correlation coefficient, which indicated that macrophages expressed SPP1 in the TME and interacted with other cell types through the SPP1–CD44 axis in the surrounding area of tumour (Figure S5F). Moreover, we observed a correlation between the abundance of SPP1^+^ macrophages and CD8^+^ T cells, CD4^+^ Tregs, as well as Th17 cells in spots, consistent with the results of SPP1–CD44 interaction in intercellular networks (Figure 4F). mIHC also indicated the co‐localisation of CD44^+^ CD4^+^ or CD44^+^ CD8^+^ T cells with SPP1^+^ CD68^+^ macrophages (Figure 4G,H). Collectively, we unveiled the differential interaction pattern of immune cells in the CRC macroenvironment, and identified SPP1–CD44 interaction as a key immunosuppressive orchestrator conducted by SPP1^+^ macrophages. These specific intercellular communications of immune cells characterised the immune profile in each location, and rewired interactions may be potential targets for ICI therapy.

### 3.5 Comprehensive analysis correlated TLS with anti‐tumour systemic immunity which was prognostical and predictable

**FIGURE 5:**
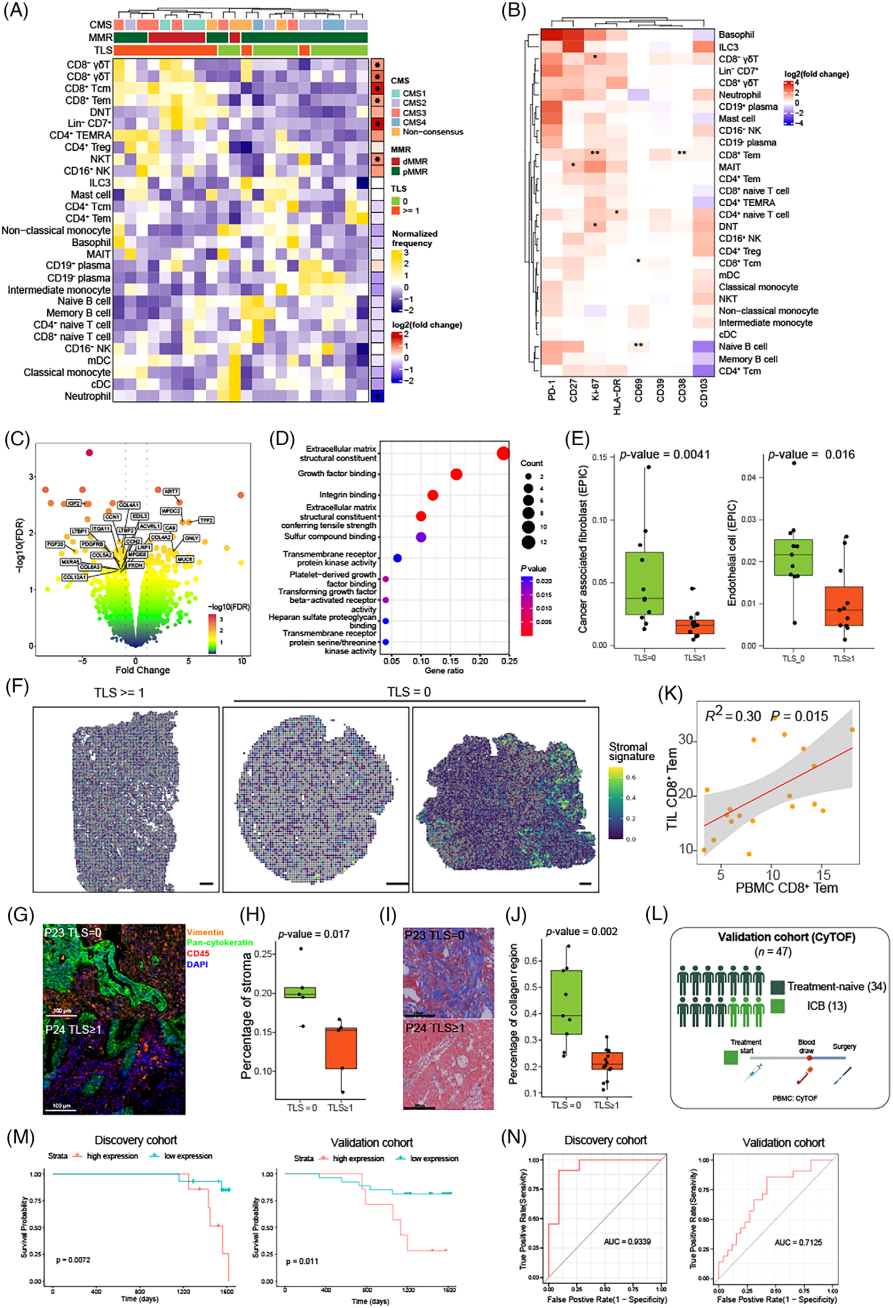
The influence of tertiary lymphoid structure (TLS) on the colorectal cancer (CRC) macroenvironment. (A) Heat map showing the frequency of each cluster in tumour with or without TLS. Arcsine transform was performed for frequency. (Left) Log2 fold‐change of proportion and significance were aligned on the right side. (*p < .05; **p < .01, likelihood ratio test. *p* values were adjusted by Benjamini–Hochberg procedure.) (B) Comparing the protein expression of each cell cluster between tumours with or without TLS. *T*‐test was performed and *p* values were adjusted by Benjamini–Hochberg procedure. (C) Volcano map of differential expression genes (DEGs). The *x*‐axis is the fold change of gene expression. Negative values indicate down‐regulation for genes in tumour with TLS when positive values indicate up‐regulation. The *y*‐axis is the minus log10 scale of the adjusted *p* values. (D) Bubble plot showing the top 10 enriched pathways for DEGs. (E) Comparisons of deconvoluted cell abundance between tumour with or without TLS based on RNA‐seq data. Distributions were compared using *t*‐test. (F) Expression of stromal signature in spots covering CRC slices with or without TLS. (G) Representative pictures of epithelial, stromal and immune cell staining in the tumour. (H) Comparisons of stromal cell percentage between tumour with or without TLS based on mIHC. Five samples per condition. *T*‐test. (I) Representative images of Masson's trichrome staining for tumour samples. (J) Bar plot showing the percentage of collagen‐positive region in the tumour slice. *n* = 9 and *n* = 14 group with no TLS and TLS, respectively. *T*‐test was performed. (K) Scatter plots show the correction of CD8^+^ Tem between peripheral blood mononuclear cell (PBMC) and the error band indicates 95% confidence interval. (L) Graphical description of the validation cohort for cytometry by time‐of‐flight (CyTOF). (M) Kaplan–Meier curves illustrating the overall survival (OS) for patients stratified by high and low expression of both PD‐1 and CD69 in CD8^+^ Tem cells from PBMC. Left, for patients in discovery cohort. Right, for patients in validation cohort. A two‐sided log‐rank test was performed. (N) Receiver operator characteristic (ROC) curves for the expression of PD‐1 and CD69 in CD8^+^ Tem cells from PBMC to distinguish CRC patients with or without TLS. AUC, area under the curve.

Since we have unveiled the general immunosuppressive macroenvironment for CRC patients, it is critical to further explain the factors that can regulate the immune macroenvironment of CRC. Intrinsic factors included atopic lymphoid structure, tumour gene expression signature, when external factors like ICI treatment are also able to alter systemic immunity. Firstly, we analysed the organisation of cellular populations which was confirmed to be altered in the tumour and surrounding regions. Spatial mapping of cells indicated that these cells were enriched at TLS (Figure S5G). TLS could occur at intra‐tumoural, peri‐tumoural and mesenteric regions, and ILF, presenting in mucosa or submucosa under physiological conditions, could be also found in the mucosal regions near to tumour (Figure S6A). We divided CRC patients into groups with or without TLS based on its presence at intra‐tumoural, peri‐tumoural and mesenteric regions, excluding ILF which could also occur in physical condition. We next asked whether this structure also modified systemic anti‐tumour immune response. Comparing the shift in the cell abundance between tumours with or without TLS showed an increasing infiltration of T cells including γδ T cells, CD8^+^ Tcm, CD8^+^ Tem, DNT and NKT cells in tumours with TLS, accompanied by a decrease in neutrophil (Figure 5A). Differential expression analysis also depicted altered functional capacity of T cells, with up‐regulated expression of Ki‐67 and CD38 in CD8^+^ Tem cells from tumours with TLS, demonstrating a higher proliferative and activated status (Figure 5B). Cellular proliferation was a potential mechanism contributing to the increased infiltration of CD8^+^ Tem cells. These results unveiled that TLS could boost anti‐tumour immunity in the TME.

To understand the different molecular features of tumours with or without TLS, we performed differential expression analysis based on RNA‐seq data to investigate enriched biological pathways. Tumours lacking TLS showed up‐regulated expression of genes related to extracellular matrix structural constituents, growth factor binding and integrin binding (Figure 5C,D). These signatures elicited us to investigate the expression of these DEGs in stromal cells, and we found that DEGs enriched in top 10 pathways were highly expressed in stromal cells including fibroblasts, myofibroblasts and endothelial cells (Figure S6B). Identification of cell status and ecosystems from bulk RNA‐seq data also demonstrated increased infiltration of stromal cells in tumours lacking TLS (Figure 5E). We defined a gene list of these DEGs as stromal signature. Analysis of stromal signature in spots reflected the increased infiltration of stromal cells in tumours without TLS (Figure 5F). Moreover, mIHC and Masson's trichrome staining also revealed increasing stromal cell counts as well as overwhelmed collagen fibres in the tumour deprived of TLS (Figure 5G–J and Tables S4 and S5). And the shift of stromal cell abundance was not contributed by altered components of epithelial cells and immune cells which was not significant shown in mIHC staining (Figure S6C,D). We hypothesised that the enrichment of stromal cells in TME could prevent the formation of TLS; however, the underlying mechanisms need further studies. Since TLS fuelled anti‐tumour immune response, CRC patients with higher TLS signature was associated with better overall survival (OS), when overexpressed stromal signature confronted patients with the risk of worse survival (Figure S6E,F).

Given the capacity of immune cells to circulate and implement their functions by migrating to the homing lesions via the bloodstream, we further investigated the association of immune response between tumour and blood samples by demonstrating their correlation of cell abundance (Figure S6G). Interestingly, the proportion of CD8^+^ Tem cells in tumour was positively related with its counterpart in the PBMC (Figure 5K). These results indicated a potential relationship between immune cells in tumours and those circulating in the bloodstream, which encouraged us to detect the shift of immune cell abundance and capacity in PBMC based on the TLS presence. Although TLS did not drove shifts in the immune cell compositions of PBMC (Figure S6H), alteration in functional capacity was recognised including the expression of PD‐1, CD27 and CD69 in T cells (Figure S6I). It was shown that PBMC from the group with TLS demonstrated a lower expression of PD‐1 and CD69 in CD8^+^ Tem cells. These results suggested the phenotype of CD8^+^ Tem cells in the PBMC was associated with the presence of TLS, potentially serving as a promising biomarker predictive of TLS in the CRC macroenvironment. To validate the potential utility of CD8^+^ Tem in the blood as a tool for prognosis and TLS prediction, 47 patients who were treatment‐naïve or received ICI therapy were recruited in validation cohort. PBMC was extracted before surgery and processed to CyTOF using our antibody panel (Methods, Figure 5L). As a result, 6 465 442 cells were recovered with 24 clusters identified (Figure S7A,B). Survival analysis showed that CD69 and PD‐1 expressed in CD8^+^ Tem from PBMC were favourable prognostic markers for treated‐naïve CRC patients (Figure 5M). Receiver operator characteristic (ROC) analysis also showed the high accuracy based on this expression pattern to distinguish treated‐naïve CRC with or without TLS in the discovery cohort (AUC = .9339; Figure 5N, left). In validation cohort, CD69 and PD‐1 expressed in CD8^+^ Tem was also able to stratify patients with TLS for treated‐naïve patients (AUC = .6917) and patients with ICI treatment (AUC = .7000, Figure S6J). Better AUC was achieved when combining these patients receiving different therapy (Figure 5N, right). These results indicated that our discovery could be applied to treated‐naïve patients for stratifying into immunotherapy, and to patients with ICI treatment for predicting TLS occurrence which was associated with better OS.

Besides PBMC, we also demonstrated the shift in immune profile of bowel wall. Minor differences were identified in immune cells from bowel wall, with notable distinctions observed in the muscularis propria (Figure S6K). Lower expression of PD‐1 and higher expression of Ki‐67 were observed in CD8^+^ Tem cells if TLS presented (Figure S6L). Collectively, our findings unveiled the influence of TLS on the CRC macroenvironment, drove by the altered organisation and functional capacity of CD8^+^ Tem cells.

### 3.6 CMS profiles CRC immune macroenvironment

**FIGURE 6:**
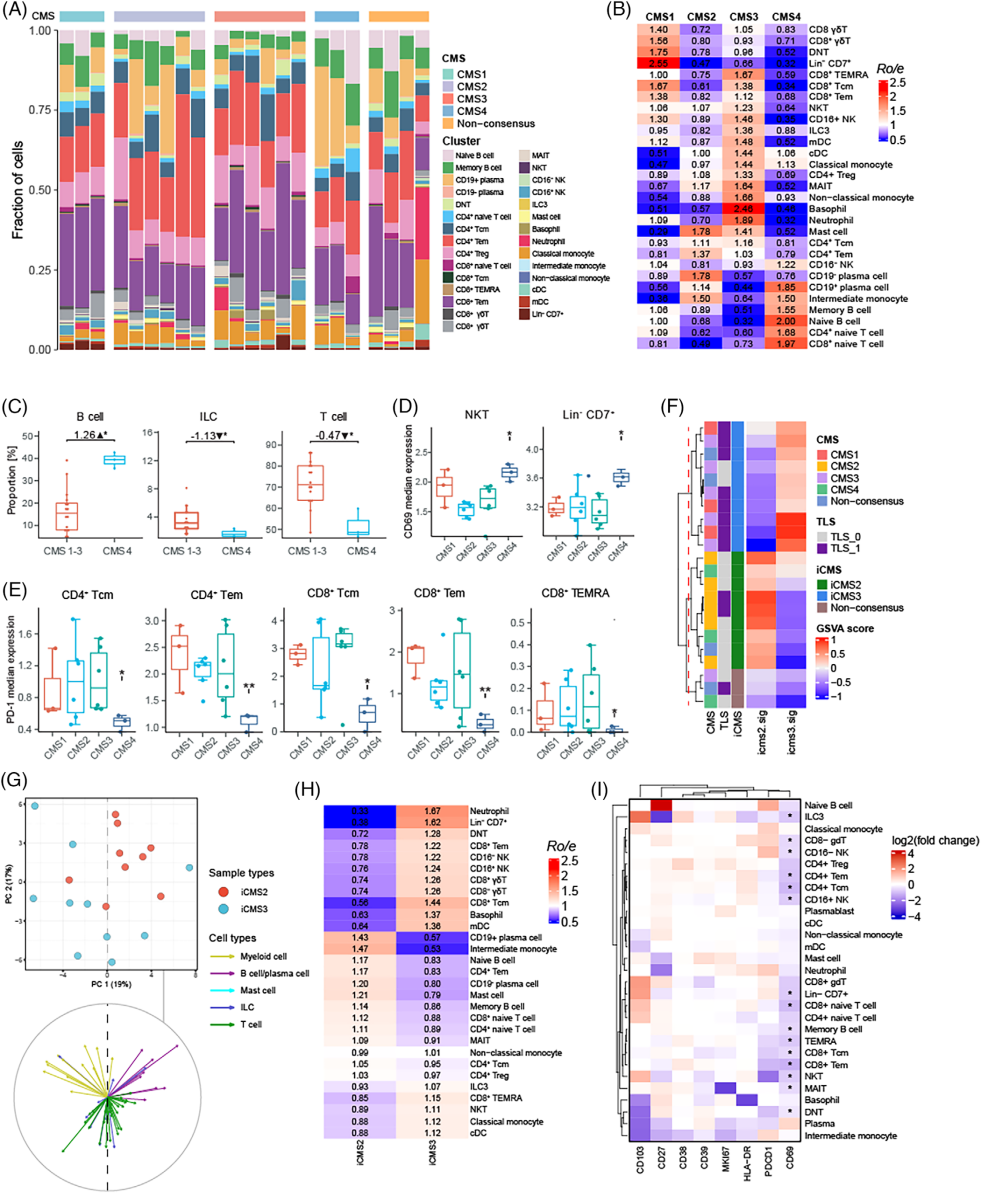
Immune profiling of the colorectal cancer (CRC) macroenvironment subclassified by consensus molecular subtype (CMS). (A) Cytometry by time‐of‐flight (CyTOF) data showing cell subset proportions of tumour samples subclassified by CMS. (B) The tissue preference of each cluster for tumour samples grouped by CMS. (C) Comparing the proportion of broad cell types in tumour subclassified by CMS. Likelihood ratio test was performed. *p* values were adjusted by Benjamini–Hochberg procedure. (D, E) Comparing the protein expression of each cell cluster between CRC with different CMS. *T*‐test was performed and *p* values were adjusted by Benjamini–Hochberg procedure. (F) Heat map showing Gene Set Variation Analysis (GSVA) results of intrinsic CMS (iCMS) gene sets in CRC RNA‐seq data. (G) Principal component analysis (PCA) of cell type composition demonstrating the distribution of iCMS2 and iCMS3 tumours (top) and associated PCA loadings (bottom). (H) The tissue preference of each cluster for tumour samples grouped by iCMS. (I) Comparing the protein expression of each cell cluster between iCMS2 and iCMS3 tumour. *T*‐test was performed and *p* values were adjusted by Benjamini–Hochberg procedure. *Ro/e*, the ratio of observed to expected cell number. Number above bracket, log2 fold‐change; ▲increased; ▼decreased; *p < .05; **p < .01.

Recent pioneering studies have revealed important features of the TME in CRC and established CMS using scRNA‐seq and bulk RNA sequencing.^27^, ^46^ These CMS classifications identify key gene expression patterns associated with CRC, such as immune activation, WNT and MYC signalling, metabolic dysregulation, stromal invasion and angiogenesis.^46^ Despite these advancements, there is still a lack of systematic studies investigating the immune profiles associated with each CMS. Our observations show that T cells expand within the human TME when TLS are present, while stromal cells can inhibit TLS formation. Based on this, we hypothesised that the presence of TLS and immune cell infiltration may be connected to the CMS classifications. Our results demonstrate that TLS are more frequently found in CMS1 and CMS3 tumours (Table S6). We performed cell abundance analysis across CMS groups and found that CD8^+^ T subsets, Lin^−^ CD7^+^ cells and DNT cells were enriched in CMS1 tumours, and that CD8^+^ T subsets and myeloid cell subsets were increased in CMS3 tumours. Conversely, CMS4 tumours were associated with higher infiltration of B cell subsets and naïve T cells, while CMS2 tumour did not displayed a distinct immune profiling (Figure 6A,B). Additionally, comparing with CMS1–3 tumours, CMS4 counterparts were composed of more B cells, less ILC and T cells (Figure 6C). Moreover, the expression of CD69 in NKT and Lin^−^ CD7^+^ cells from CMS4 tumours was remarkably higher than their counterparts in CMS1–3 tumours, while the expression levels of PD‐1 in CD4^+^ Tcm, CD4^+^ Tem, CD8^+^ Tcm, CD8^+^ Tem and CD8^+^ T_EMRA_ from CMS4 tumours were significantly lower than their counterparts from other CMS groups (Figure 6D,E). These results revealed that the lower abundance of T cell, lower expression of PD‐1 and higher infiltration of B cell in CMS4 tumours, suggesting a potential mechanism of resistance to anti‐PD‐1 therapy in this subtype of CRC. Furthermore, we investigated the composition of immune cells in PBMC according to CMS groups. B cell/plasma cell subtypes and γδ T cells were increased in PBMC from CMS4 patients, indicating a potential relation of B cell/plasma cells between tumour and blood in these patients (Figure S8A).

Recently, two inherent malignant epithelial subtypes were identified for CRC by single‐cell and bulk transcriptomic analyses, which reported a refined CMS classification of CRC.^27^ iCMS classifies CRC based on intrinsic epithelial subtypes, MSI status and the presence of fibrosis. MSI‐H CRC and one‐third of microsatellite‐stable (MSS) CRC were classified into iCMS3, which represented an activated anti‐tumour immunity phenotype. iCMS3_MSS was reported to show increasing infiltration of T and myeloid cell and anti‐tumour cytotoxicity, similar to MSI CRC. Since immune profiling of TME was only demonstrated by bulk RNA‐seq in previous study, we investigated immune cells in different iCMS macroenvironment based on our large‐scale flow cytometry data. First of all, we found that the subclasses of CRC could be successfully identified according to the gene sets of iCMS2 and iCMS3 (Figure 6F). Additionally, we found the presence of TLS was more significant in iCMS3 tumours than iCMS2 tumours, indicating that the activated anti‐tumour immune response in iCMS3 tumours could be fuelled by TLS (Table S7). Next, PCA of cell abundance uncovered a clear separation between these iCMS groups, and PCA loadings indicates that T cells, ILC and B/plasma cells contributed to the observed differences (Figure 6G). Further estimation indicated CD8^+^ T cell subsets including Tem, Tcm, γδ T cells, NK cells and Lin^−^ CD7^+^ cells were enriched in iCMS3 tumours, whereas B/plasma cell and naïve T cells were enriched in iCMS2 tumours (Figure 6H). These results indicated that the TME of iCMS3 tumours were characterised by increasing CD8^+^ T/ILC cell subtypes which were mainly associated with TLS. And enriched naïve T cells in iCMS2 tumour suggested T cell abnormal differentiation.

Finally, we explored the changes of immune cell compositions in the bowel wall as well as PBMC for iCMS groups. Minor differences were found in the abundance of cell types for the epithelial layer, lamina propria, submucosa and muscularis propria (Figure S8B). However, the functional capacity of cells in PBMC was significantly altered, showing a decreasing expression of CD69 (Figures 6I and S8C). ROC analysis confirmed that the iCMS groups could be well recognised by the expression of CD69 based on our discovery cohort data (AUC = .8444; Figure S8D). Given that the transcriptomic features of iCMS3_MSS tumours were reported to be more similar to MSI‐H colon cancers than to iCMS2_MSS tumours, iCMS3_MSS tumours have the potential to respond to immunotherapy. Therefore, our results from multi‐omics analyses characterised CD69 as a promising biomarker in blood to predict iCMS for CRC patients, which provided a new method to select iCMS3 patients for ICI treatment.

### 3.7 The alteration of CRC macroenvironment after ICI treatment

**FIGURE 7:**
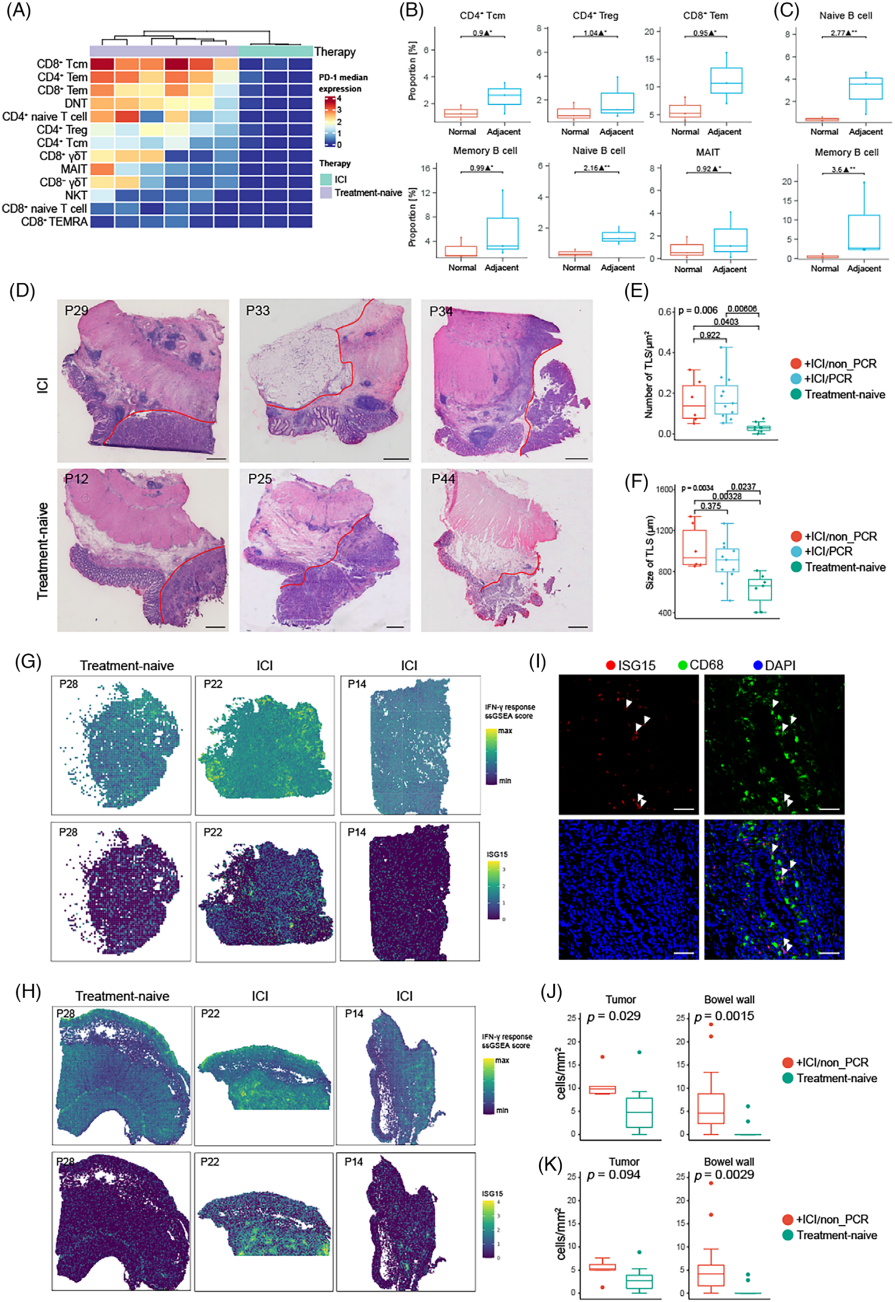
Immune profiling of the macroenvironment in colorectal cancer (CRC) patients with immune checkpoint inhibitor (ICI) treatment. (A) Heat map showing expression of PD‐1 in T cell subsets from tumours with or without anti‐PD‐1 therapy. (B, C) Comparison analysis for the proportion of CD45^+^ clusters between normal bowel and adjacent bowel in lamina propria (B) as well as muscle propria (C) from CRC patients with ICI. *n* = 3 samples per condition. Likelihood ratio test was performed. *p* values were adjusted by Benjamini–Hochberg procedure. (D) Haematoxylin and eosin (H&E) staining images showed peri‐tumoural tertiary lymphoid structure (TLS). Tumor margin was indicated by red lines. (E, F) Quantification of the number (E) and size (F) of TLS among patient groups. One‐way analysis of variance (ANOVA) test and Tukey's post hoc tests were performed. (G, H) A module score of interferon‐γ (IFN‐γ) response (top) as well as expression of ISG15 (bottom) overlaid in spatial transcription (ST) spots of tumour slides (G) and normal bowel slice (H). (I) Immunofluorescence images of tumour sections from the ICI group. White arrows point to cells positive for both ISG15 and CD68. (J) Quantification of the density of ISG15^+^ cells in tumour sections (left) or bowel wall sections (right) with or without ICI therapy. Wilcoxon test was performed. (K) Quantification of the density of ISG15^+^ CD68^+^ macrophages in tumour sections (left) or bowel wall sections (right) with or without ICI therapy. Wilcoxon test was performed.

ICI treatment has provided a long‐term clinical benefit for patients with several tumour types. The presence of TLS has been shown to be associated with improved objective response rate, progression‐free survival and OS.^52^, ^53^ To further understand the relationship between ICI treatment and TLS in the tumour macroenvironment, we performed single cell and spatial analysis to investigate the different phenotypes of immune cells and biological features of TLS between patients with or without ICI treatment. Anti‐PD‐1 therapy systematically inhibited the expression of PD‐1 in T cells from samples including tumour, blood and bowel wall (Figures 7A and S9A,B) and significant down‐regulation of CD39 and CD69 in ILC and T cell subsets in TME was also observed (Figure S9C,D). Minor alterations in cell abundance were observed in the normal bowel of patients after ICI treatment (Figure S9E,F). Next, we analysed the shift in the organisation of immune cells in the macroenvironment, showing increased NKT cells in blood (Figure S9G). In the tumour–adjacent bowel wall, T cell subsets including CD4^+^ Tcm, CD4^+^ Treg, CD8^+^ Tem and B cell subsets composed of memory B cell, naïve B cell and MAIT cells were significantly increased in the lamina propria (Figure 7B), when naïve B cell and memory B cell were enriched in the muscularis propria (Figure 7C). Moreover, these cellular populations were also enriched in the region of TLS, indicating that TLS contributed to the shift of cell compositions in the tumour macroenvironment (Figure S5G). Next, we compared the density and size of TLS in the macroenvironment between tumours with or without ICI treatment (Table S8). As a result, H&E staining images of tumour margin demonstrated that the density of TLS is increased after ICI therapy, with the size of TLS also significantly enlarged, for both patients with or without pCR (Figure 7D–F). Taken together, ICI treatment could promote the formation of TLS and result in increasing T cell and B cell subsets in the surrounding areas of tumour.

We further characterised molecular features of tumour macroenvironment to elucidate the anti‐tumour mechanisms of ICI treatment as well as immune escape. Gene set enrichment analysis (GSEA) showed interferon‐γ (IFN‐γ)‐response pathway was enriched in the stromal region of samples with ICI treatment (Figure S10A). IFN‐γ is a pleiotropic cytokine with both pro‐tumour and anti‐tumour activities. IFN‐γ is produced by effector CD8^+^ T cells, Th1 CD4^+^ T cells, NK and NK T cells. Nearly, all cells can response to IFN‐γ; however, the consequential reactions vary. Spatial feature plots showed the activation of IFN‐γ response pathway in the stromal region of samples including tumours or bowel walls, especially for patients with ICI treatment (Figure 7G,H). These results indicated hyperactivation of the IFN‐γ response was not only limited to the TME but also occurred systematically, although the cell composition was not significantly altered as revealed by CyTOF. This suggested that the functional capacity, rather than the composition, of immune cells was altered. As for genes enrolled in IFN‐γ response, ISG15 was highly expressed in tumours with ICI treatment (Figure 7G,H). We further investigated the expression of ISG15 by scRNA‐seq which was performed to tumour or normal bowel samples of patients with ICI treatment from our cohort.^54^ ISG15 was highly expressed in myeloid cells and T cells, especially for patients without pCR (Figure S10B). Next, we investigated the influence of IFN‐γ to myeloid cells. Re‐clustering myeloid cells identified a new macrophage group marked by a higher expression of ISG15 (Figure S10C,D). Gene ontology analysis also depicted an enriched pathway about the response to IFN‐γ (Figure S10E). Interestingly, inflammation‐associated genes such as IL2RG, TNF, IFNGR2, S100A8 and S100A9 were highly expressed in ISG15^+^ macrophages, indicating a proinflammatory phenotype (Figure S10F). We further analysed inflammatory signatures in the ST data of ICI‐induced colitis.^55^ It was shown that ISG15 was up‐regulated in the mucosa of patients with ICI‐induced colitis (Figure S10G). We also performed mIHC to confirm the expression of ISG15 in the tumour and normal bowel wall (Figure 7I). Increasing ISG15^+^ cells were detected in both the tumour region and normal bowel wall of patients with partial response for ICI treatment (Figure 7J), whereas ISG15^+^ CD68^+^ macrophages were only enriched in the normal bowel wall, indicating their proinflammatory phenotypes in the bowel wall (Figure 7K). However, ISG15^+^ CD68^+^ macrophages were not increased in tumours with ICI treatment when compared to treated‐naïve tumours, suggesting they were not dominant in the IFN‐γ response in the TME. Subsequently we investigated the expression of ISG15 in T cell subsets (Figure S10H,I). CD4^+^ GZMK^+^ T cells, CD4^+^ Tregs, CD8^+^ Tex cells, CD8^+^ ITGA1^+^ T cells and MAIT cells were found to have remarkably high expression levels of ISG15 for patients with partial response, suggesting that increased expression of ISG15 was associated with worse therapeutic efficacy of ICI treatment. OS analysis suggested a worse prognosis for CRC patients with high expression of ISG15 (Figure S10J). Analyses of transcriptomes of pretreatment melanoma biopsies from a prospective cohort of melanoma patient with ICI treatment showed that ISG15 was up‐regulated in tumours with poor response to ICI therapy (Figure S10K).^56^ ROC analysis also recognised ISG15 as a promising biomarker predictive of response to ICI treatment (AUC = .7640, Figure S10L). Taken together, ICI treatment contributed to systematic IFN‐γ response by facilitating the formation of TLS and boosting the expansion of CD8^+^ T cells, and ISG15 was identified as a proinflammatory marker which was also associated with poor response to immunotherapy.

## 4 DISCUSSION

Due to traditional sequencing technologies limited by resolution and the lack of anatomical dissection approaches, systematic anti‐tumour response to CRC has not been well elucidated. The definition of tumour immune macroenvironment underscores profiling immunity of multiple organs to investigate the dynamic shift in the organisation and functional capacity of cells, which provides a panorama of activation and suppression of immune cells and indicates available biomarkers for therapeutic targets or predictive markers. In previous studies, tumour‐burdened macroenvironment was investigated using mouse model, while the immune macroenvironment in human body was poorly defined.^19^ Moreover, since ICI treatment has provided wide clinical benefits for dMMR CRC patients despite unsatisfied therapeutic efficacy for pMMR patients, investigating the immune macroenvironment also contributes to an increased understanding of the mechanisms of ICI and developing more precise treatments.

In this study, we create a comprehensive panorama of immune macroenvironment in CRC patients, unveiling the baseline and associated perturbation of the integrated immune profile. Human colorectal carcinoma originates from epithelial cells of intestine. As tumour cells invade epithelial layer, lamina propria, submucosa and muscularis propria step by step in situ during tumourigenesis, they encounter different immune cells resident at each layers deliver an anti‐tumour response by proliferation and migration, when immune cells in blood also function through circulation. The phenotypes of immune cells in the mucosa and tumour have been well clarified; however, few efforts have been made to properly define their properties in different sites and the associated alterations related to tumours. Facilitated by a new dissection method, our study enrolls 10 locations for each CRC patient, including tumour, blood, 4 layers of normal bowel, as well as tumour–adjacent bowel, to construct a landscape of immune macroenvironment. Here, we show a distinct immune profile of the human bowel, describing the distribution of immune cells in each layer at single‐cell resolution as well as in the spatial context. Generally, epithelial layer, lamina propria, submucosa and muscularis propria of the human bowel wall are characterised by enriched CD8^+^ T cells, plasma cells, B cells/naïve T cells and myeloid cells, respectively. The functional capacity of resident cell types is associated with their spatial context. For example, CD39^+^ CD8^+^ IELs in the epithelial layer have the ability to maintain immune homeostasis and ameliorate disease activity of Crohn's disease.^57^ ILFs were structures specifically situated in the lamina propria or submucosa linked to GC‐based B cell priming, with ILFs in the submucosa serving as important adaptive immune‐inductive sites for the colon lamina propria based on IgA repertoire.^30^ The macrophages resident in the muscularis propria were capable of interacting with neurons and regulating the movement of large bowel.^58^, ^59^ Collectively, these results provide important insights into the spatial organisation of immune cell types in the human large bowel.

When CRC occurs and progresses in situ, pro‐tumour and anti‐tumour immunity are programed in the macroenvironment, including the TME, surrounding layers of the bowel wall and blood. Immune profiling of TME has been widely examined in previous studies and associated adaptive immune‐inductive features have been recognised.^16^, ^60^ Here, we provide important insights into the spatial immunity of CRC, including spatial cell abundance, capacity and global cell–cell interactions. First of all, we identify an immunosuppressive environment established by increased Tregs in the tumour, lamina propria and muscularis propria. Both CD39 and PD‐1 are immunoinhibitors and expressed at higher level in T cell subsets. Moreover, CD39 is up‐regulated in Tregs from all layers of the tumour–adjacent bowel wall as well as tumour tissue, indicating its pro‐tumour role in the peri‐tumoural or intra‐tumoural regions during CRC progression. CD39 is an enzyme encoded by ENTPD1 gene, which converts adenosine triphosphate into adenosine diphosphate and cyclic adenosine monophosphate, thereby leading to the immunosuppressive environment caused by the accumulation of adenosine.^61^ Therefore, CD39 is a promising new target for ICI therapy. However, CD39 is also expressed in CD8^+^ T cells and γδ T cells located in the epithelial layer, and plays an important role in mucosal immune homeostasis. A decreased frequency of CD39^+^ γδ T cells was observed in patients with inflammatory bowel disease compared with healthy controls, which was further supported by the suppression of CD39 expression in Crohn's disease by endogenous antisense.^57^, ^62^ Therefore, blockade of CD39 may have a potential risk of ICI‐induced colitis. Next, integrated analysis of ligands and receptors in all sites identify local immune regulation, which can be used to define the immune profile. As shown, pathways of PD‐L1, GP1BA, RESISTIN and SPP1 are exclusively activated in tumour. A previous study indicated that PD‐L1 was expressed in the tumour but not in adenoma, suggesting that the interaction of PD‐1 and PD‐L1 is essential for the establishment of an immunosuppressive TME.^63^ Moreover, we find that SPP1 is only expressed in macrophages derived from tumour, which mainly exert effect on T cell subsets. Mapping cells and cell–cell communications in situ shows this unique interaction in the TME and validates the co‐localisation of SPP1^+^ macrophages with CD8^+^ T cells, CD4^+^ Treg cells, as well as Th17 cells. There were studies which reported SPP1^+^ macrophages could interact with tumour cells and other immune cell types, leading to an immunosuppressive microenvironment.^16^, ^64^, ^65^ Our study revealed that the SPP1–CD44 interaction is essential in regulatory hubs of tumour.

Our results not only identify adaptive immune response in the CRC macroenvironment but also show the importance of adaptive immune‐inductive structure, such as TLS, locating at the tumour–adjacent bowel wall. T cell and B cell subsets make up the TLS components, and they are enriched in the tumour–adjacent bowel wall within 2 cm away from the tumour margin. The presence of TLS in the CRC macroenvironment fuels anti‐tumour immune responses by increasing the infiltration of CD8^+^ Tcm, CD8^+^ Tem, γδ T cells and NKT cells in TME. Additionally, the up‐regulated expression of CD38 and Ki‐67 reveals an activated and proliferative status of CD8^+^ Tem cells. CD8^+^ Tem cells are an effector phenotype marked by granzyme production and have the ability to kill tumour cells. TLS functions as a station for the expansion and activation of T cells. We also identify fibrosis in the TME as an important factor that prevents the formation of TLS. Given that TLS provides a new link between the tumour and the immune system, it can exert influence to immune cells in surrounding organs such as bowel wall and peripheral blood. The function capacity of T cells in PBMC and the tumour–adjacent muscularis propria is altered in patients with TLS. The frequency of CD8^+^ Tem cells in PBMC is positively correlated with that of the tumour. These results unveil a relationship between PBMC and tumour for CD8^+^ Tem cells. Furthermore, the expression of PD‐1 and CD69 in CD8^+^ Tem cells is capable to identify CRC with or without TLS. Since PBMC is an ideal liquid biopsy approach to evaluate systematic immunity, assessing the expression of PD‐1 and CD69 in CD8^+^ Tem cells by cytometry is accessible, while the tissue biopsy is not able to represent the integrated immune macroenvironment. Collectively, in this part our approach and findings indicated the application of blood test to stratify patients into ICI treatment.

We also elucidated the association between tumour molecular features and TLS presence. The iCMS classification is a dichotomy of molecular subtypes of CRC. iCMS3 tumours contribute to the increased presence of TLS in the macroenvironment, leading to an enrichment of CD8^+^ T cells and ILC. Strikingly, different iCMS tumours also exert different effect on PBMC. Lower expression of CD69 is observed in most cell types in PBMC from iCMS3 patients. These results indicate that CD69 can serve as a biomarker to categorise CRC into iCMS2 or iCMS3 groups, and provide new insights into systemic immunity alteration determined by CMS.

ICI treatment provides clinical benefit to some patients, although the hallmarks of treatment response have not been clearly elucidated. Assessing the genetic profile of tumours and the systematic activity of host's immune system are essential to guide treatment decisions. This work also unveils the alteration of immune macroenvironment in patients with anti‐PD‐1 therapy. There is an increase in the number of TLS occurring in the peri‐tumour regions, and the size of TLS also enlarges after ICI treatment, for patients with or without pCR. In our cohort, even dMMR patients without pCR also experienced a partial shrinkage of their tumours, indicating therapeutic efficacy of ICI. Given that the presence of TLS could predict ICI efficacy, the new approach to detect TLS based on PBMC helps measure treatment response and make treatment decision for CRC patients.^53^ ICI treatment also contributes to a systematical IFN‐γ response, which results from an increasing proportion of CD8^+^ T cells as well as cytotoxicity. While IFN‐γ response is essential in the adaptive immune response, current evidence suggests that it may exert a negative effect on CD8^+^ T cell responses. The IFN‐γ‐associated gene, ISG15, is up‐regulated in CD8^+^ T cell subsets from non‐pCR patients, contributing to resistance to ICI treatment. Higher expression of ISG15 in CRC confronts patients with worse survival rate, and ISG15 is also up‐regulated in melanomas that are not sensitive to ICI treatment. Moreover, ISG15 is also expressed in macrophages located within bowel wall, indicating a proinflammatory capacity. It is shown that ISG15^+^ macrophages increase in the bowel wall of patients with ICI therapy, although morphology revealed by H&E staining dose not show inflammation. In the bowel samples from patients with ICI‐induced colitis, the expression of ISG15 is also significantly increased in mucosa, indicating that IFN‐γ triggers inflammation by macrophages. These results show a systematical IFN‐γ response following ICI treatment, and ISG15 plays an important role in therapy resistance and inflammation within the bowel wall during ICI therapy. In general, the final part of this study evaluates the influence of ICI therapy to macroenvironment, providing more evidence of positive regulation by TLS, and therefore detection of TLS by blood test is a promising approach to help clinical decision for CRC treatment.

Our study utilised cutting‐edge technology and a new anatomical dissection method to unveil the features of CRC immune macroenvironment, providing a detailed, high‐resolution stereoscopic map of anti‐tumour immunity and also discovering biomarkers for development of clinical approach in stratifying ICI treatment response. However, this study has some limitations. The first limitation is the small number of patients in the cohorts. And treatment‐initiated biopsy was not enrolled, which could help unveil the regulation of immune macroenvironment. Although this study provides considerable source for learning immune macroenvironment including transcriptomics and proteomics like RNA‐seq and CyTOF, the number of samples processed by scRNA‐seq and ST‐seq is limited.

## 5 CONCLUSIONS

In summary, our study elucidated an integrated immune profiling of the CRC macroenvironment, interpreting the organisation, systematical activation and suppression of immune cells. These findings provide important insights into the overall immune response in patients with different CMS and present promising biomarkers for the development of precise immunotherapy strategies for CRC patients.

## AUTHOR CONTRIBUTIONS

Ping Lan, Xianrui Wu and Kui Wu conceived the study. Haoxian Ke, Peisi Li, Zhihao Li and Xian Zeng designed the experiments as well as analysis and wrote the manuscript. Shuzhen Luo, Xiaofang Chen, Xinlan Zhou and Shichen Dong optimised and performed the scRNA‐seq and Stereo‐seq experiments. Shubiao Ye and Tuo Hu optimised the analysis methodology and analysed the data. Chi Zhang, Shaopeng Chen, Junfeng Huang, Ming Yuan and Runfeng Yu provided and prepared the CRC samples. Ping Lan, Xianrui Wu and Kui Wu reviewed and edited the manuscript. Ping Lan, Xianrui Wu and Kui Wu provide resources supporting this study. Ping Lan, Zhonghui Tang and Xianrui Wu provided advice for the experiments of the study. Ping Lan, Xianrui Wu and Kui Wu supervised the project.

## CONFLICT OF INTEREST STATEMENT

The authors declare no conflicts of interest.

## ETHICS STATEMENT

This study was approved by the ethical committee of the Sixth Affiliated Hospital, Sun Yat‐Sen University (E2021084). Written informed consent was provided by all patients.

## Supporting information



Supporting Information

## Data Availability

The processed public datasets were download form Gene Expression Omnibus including GSE78220.^56^ GSE210037.^55^ GSE205506.^54^ The raw counts of The Cancer Genome Atlas (TCGA) COAD RNA‐seq data were downloaded by R package TCGAbiolinks. A ST dataset of a stage‐II CRC tissue was downloaded from 10X Genomics website, which was generated by the Visium Gene Expression Library (T1T2‐E8). And ST of the margin of CRC were downloaded from a related study.^29^ CODEX data of colon were downloaded in HuBMAP website.^48^ The data reported in this paper have been deposited in the OMlX, China National Center for Bioinformation/Beijing Institute of Genomics, Chinese Academy of Science.^66^, ^67^ (https://ngdc.cncb.ac.cn/omix/submitList, OMIX005985 for ST, OMIX005983 for RNA‐seq, OMIX005984 for scRNA‐seq and OMIX005986 for CyTOF).
