# Supplementary material for: Immune profiling of the macroenvironment in colorectal cancer unveils systemic dysfunction and plasticity of immune cells
Source: Clin Transl Med. 2025 Feb 11;15(2):e70175. doi: 10.1002/ctm2.70175 (PMC11813809; doi:10.1002/ctm2.70175)
Supplement: Supplementary file 1 — Supporting Information [file CTM2-15-e70175-s001.docx]

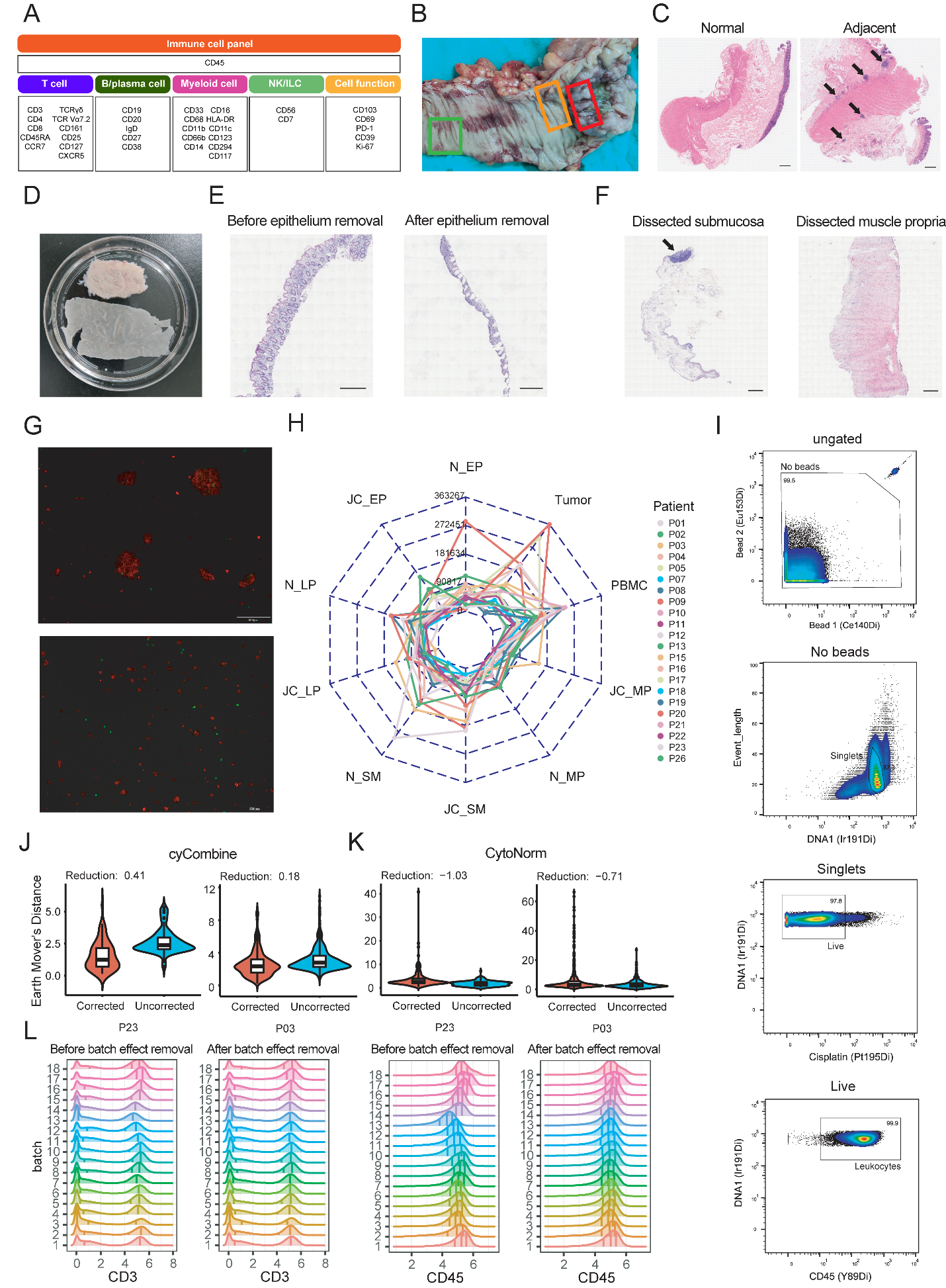


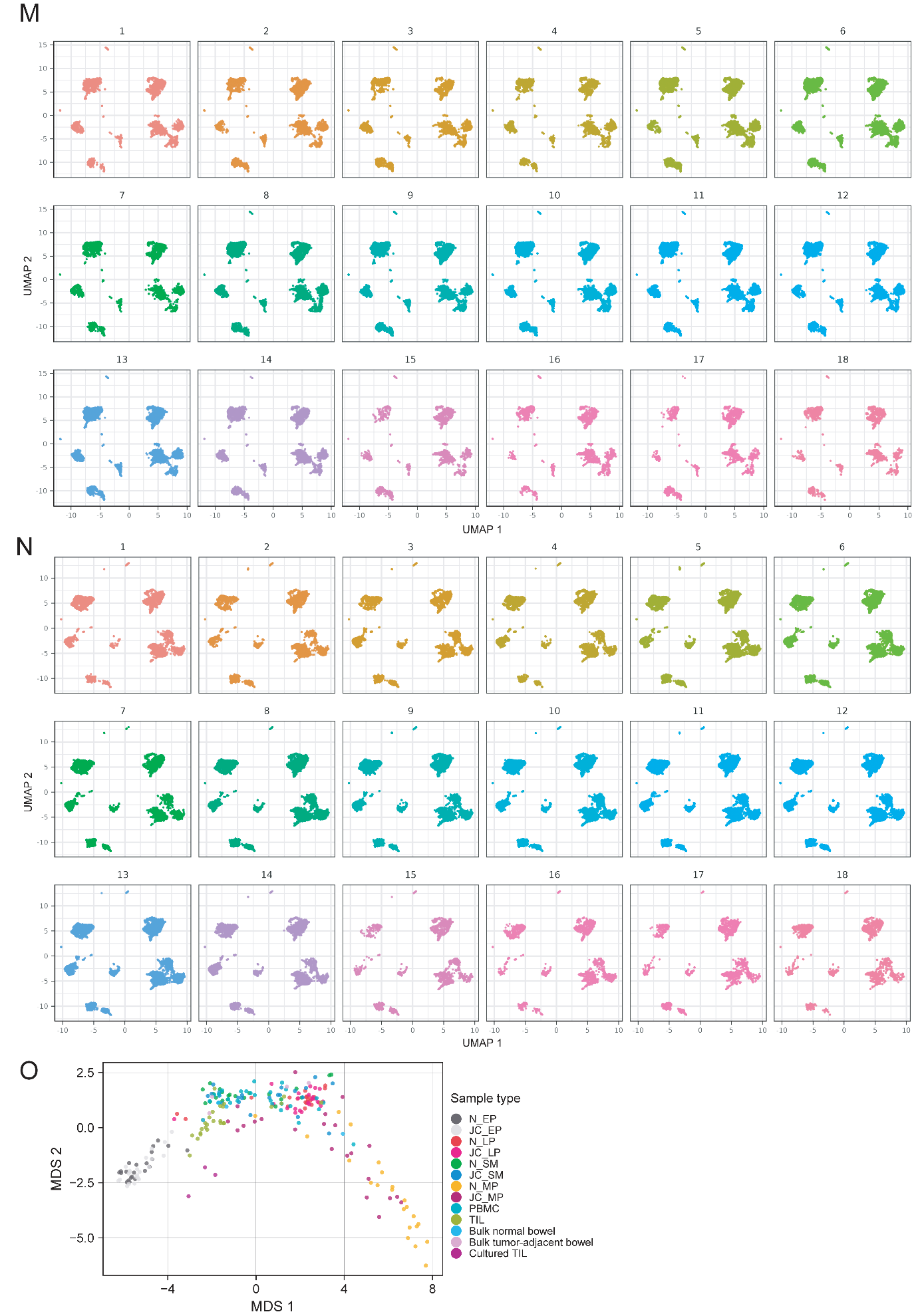


**Figure S1.** **(A)** The panel schematic depicting the lineage and functional targets interrogated by CyTOF. **(B)** Graphic description of tissue sampling. Red, orange, and green rectangle indicated sampling of tumor, adjacent bowel, and normal bowel, respectively. **(C)** H & E staining images of normal bowel (left) and adjacent bowel (right). Scale bar, 500 μm. **(D)** Anatomical dissection of mucosa and submucosa. Upper mass and lower mass indicated submucosa and mucosa. **(E)** H & E staining images of mucosa before and after removal of epithelium. Scale bar, 500 μm. **(F)** H & E staining images of dissected submucosa and muscle propria. Arrow indicated an isolated lymphoid follicle. Scale bar, 500 μm. **(G)** Immune fluorescent images of a epithelial-layer sample with EPCAM^+^ epithelial cells (red) and CD45^+^ immune cells (green). Scale bar, 200 μm. **(H)** Radar plot showing cell counts of 220 spatial samples from CyTOF experiment. **(I)** Gating strategy for identifying live CD45^+^ immune cell population in CyTOF data. **(J& K)** Violin plots showing Earth mover’s distance of clusters for data corrected by cyCombine or uncorrected data, when the reduction score of Earth mover’s distance was calculated (J). CytoNorm was tested with the same procedure (K). Data of 2 patients including P23 and P03 was used. **(L)** The expression of CD3 or CD45 across each batch before and after batch effect removal. **(M & N)** UMAP plots of cells equally sampled from each batch before (M) and after (N) batch effect removal. **(O)** MDS plots showing 240 spatial samples before and after batch effect removal. N, normal, JC, juxta-cancerous; EP, epithelial layer; LP, lamina propria; SM, submucosa; MP, muscularis propria. TIL, tumor infiltrated lymphocyte. eTIL, TIL sample which was cultured and expanded *in vitro* for 5 days.


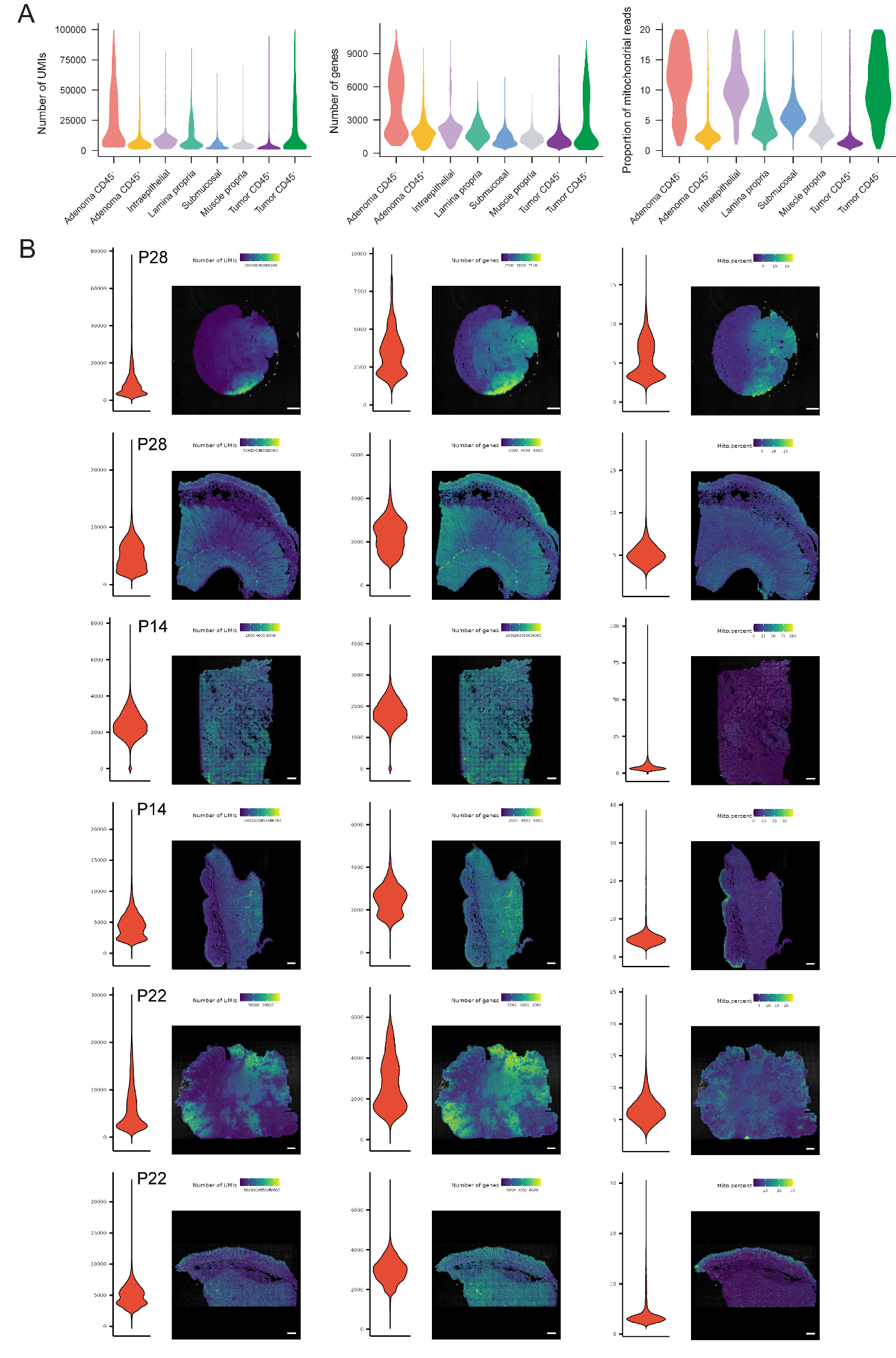


**Figure S2.** **(A)** The number of UMI counts, gene features, and mitochondrial gene percentages shown for scRNA-seq data. **(B)** The number of UMI counts, gene features, and mitochondrial gene percentages shown for ST-seq data.


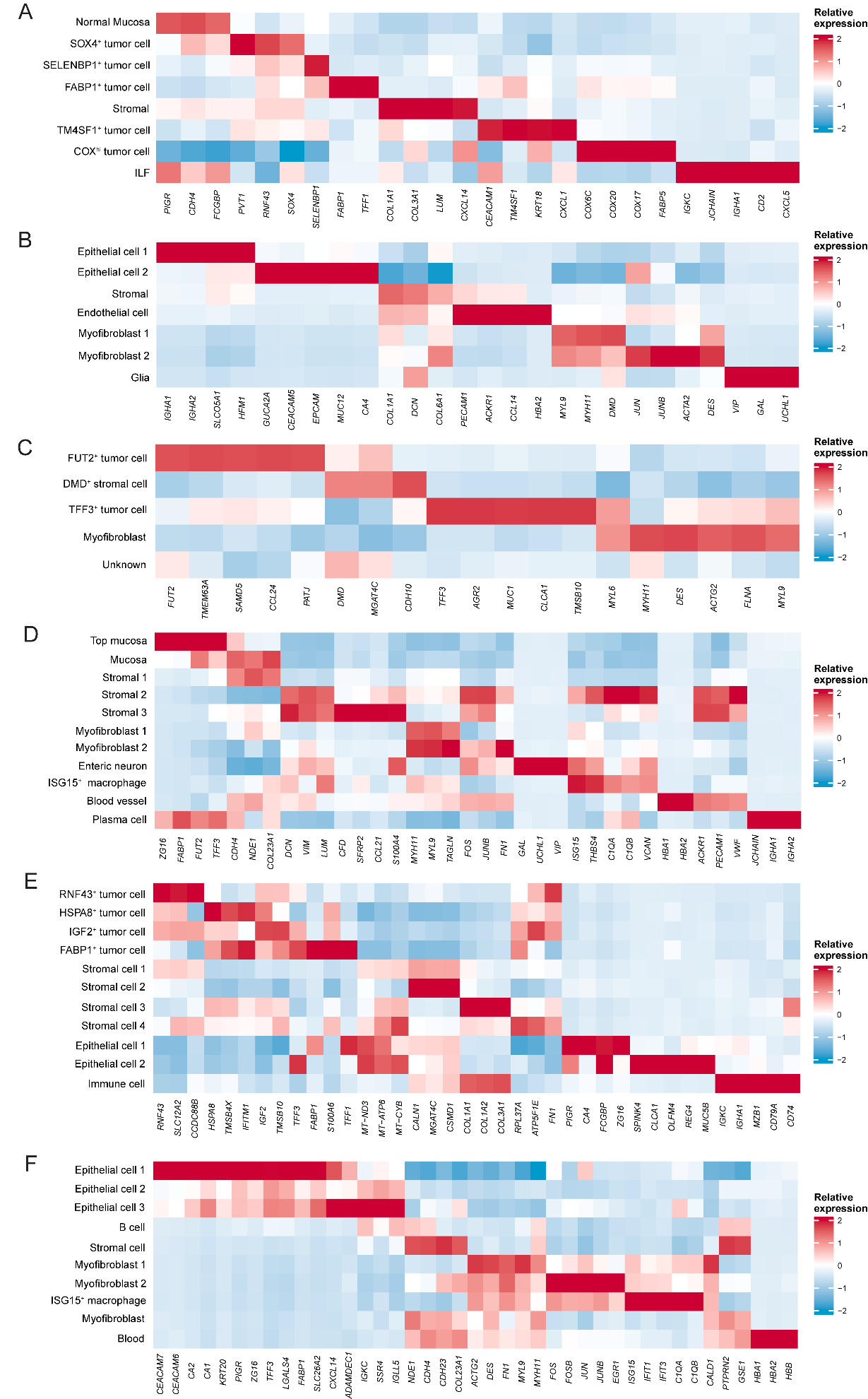


**Figure S3.** Heat maps showing unique markers of clusters identified in ST-seq data corresponding to Figure 1F.


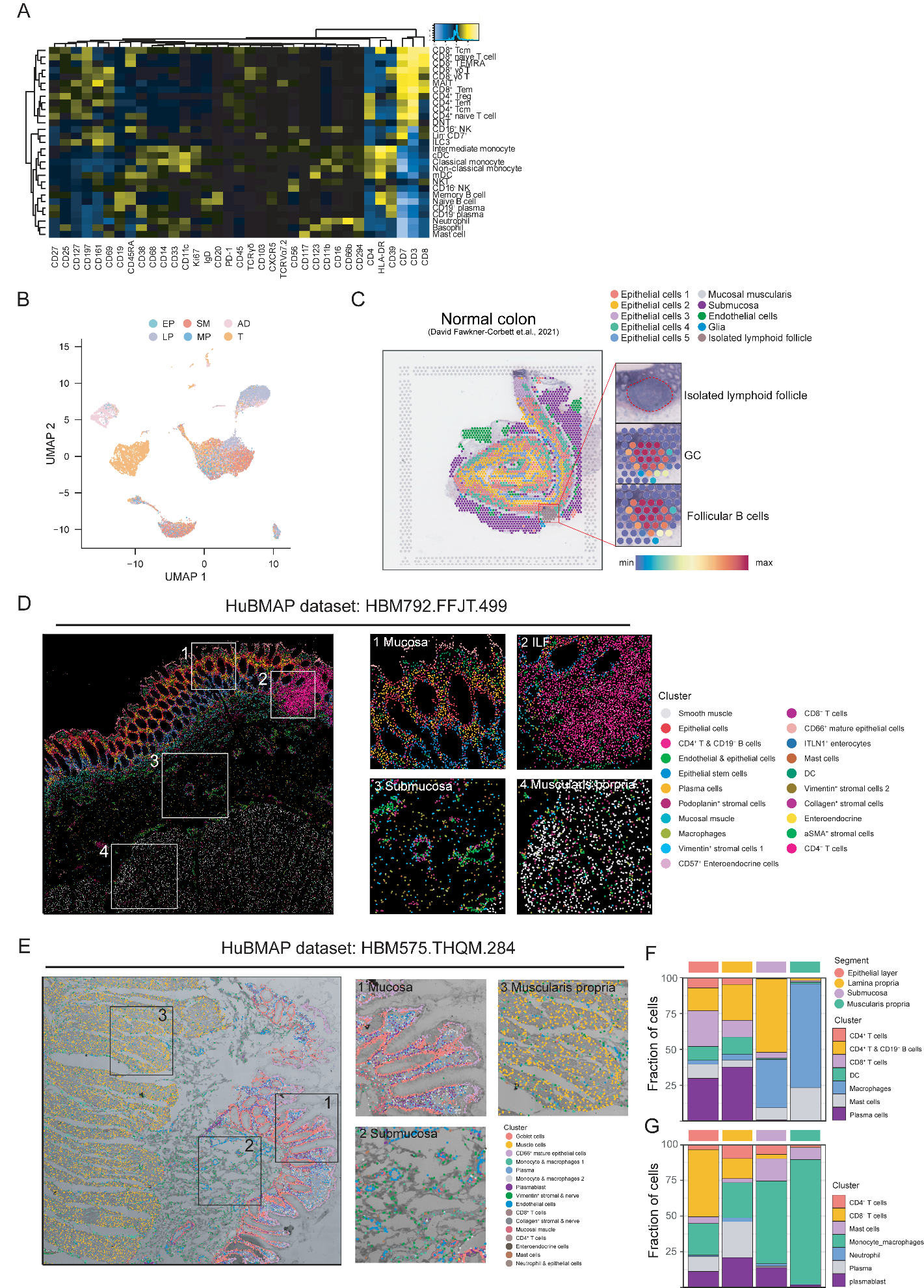


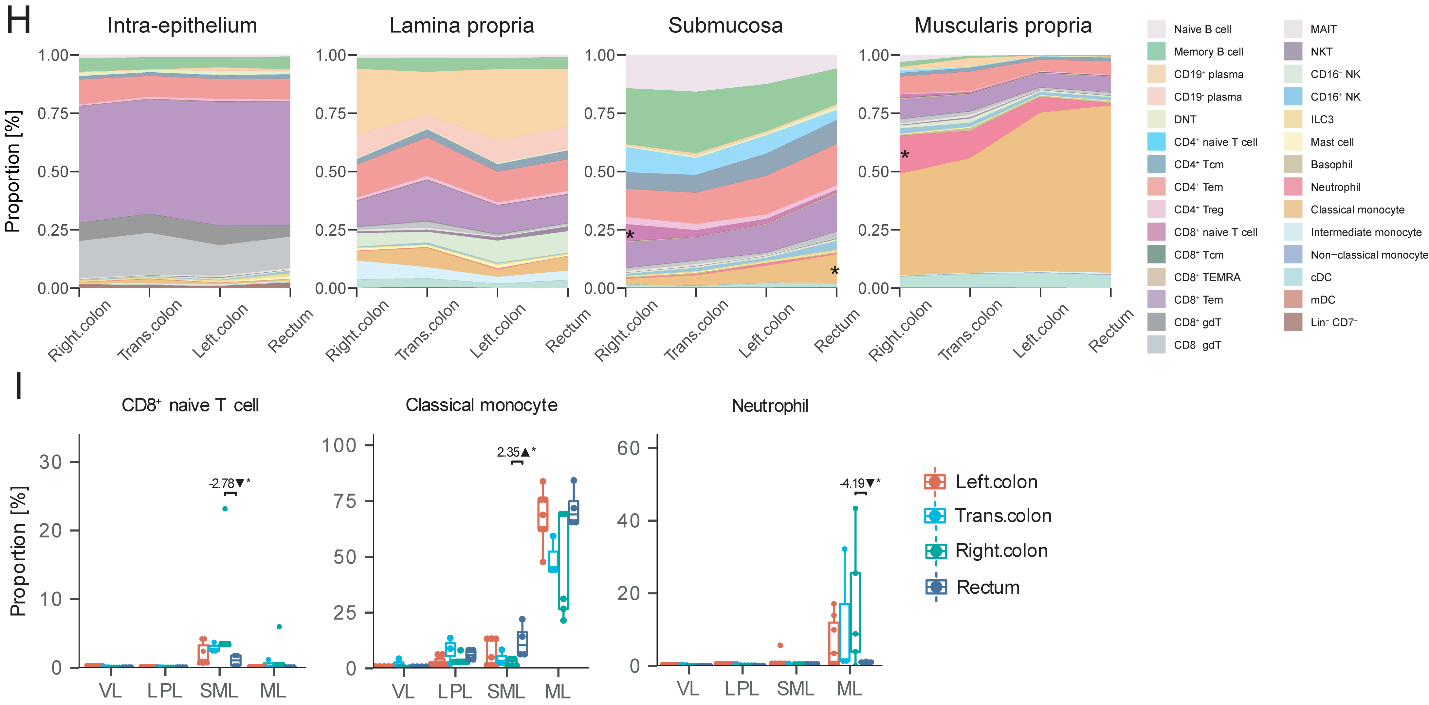


**Figure S4.** **(A)** Heat map of MEM scores of 35 markers of 29 clusters. **(B)** UMAP plots of 25973 cells indicating locations. EP, epithelial layer; LP, lamina propria; SM, submucosa; MP, muscularis propria. AD, adenoma. T, tumor. **(C)** Clusters were visualized on a normal bowel tissue from published data (left). Deconvoluted B cell abundance in spots covering submucosal lymphoid follicle was indicated by cell2location algorithm (right). **(D & E)** CODEX images showing distribution of cell populations in colon slices. Representative FoVs were also indicated. **(F** **& G)** Bar graphs of proportion of cellular subtypes in epithelial layer, lamina propria, submucosa, and muscularis propria. Figure F and G was corresponding to D and E, respectively. **(H)** Relative proportions of cell type at epithelial layer, lamina propria, submucosa, and muscularis propria across different bowel segments. **p* < 0.05, likelihood ratio test. *p* values were adjusted by Benjamini-Hochberg procedure. **(I)** Significantly differential abundance of cell types between regions in each layer. Right colon: 5 samples; transverse colon: 3 samples, right colon: 7 samples; rectum: 4 samples; number above bracket, log2 fold-change; ▲increased; ▼decreased; **p* < 0.05, likelihood ratio test. *p* values were adjusted by Benjamini-Hochberg procedure.


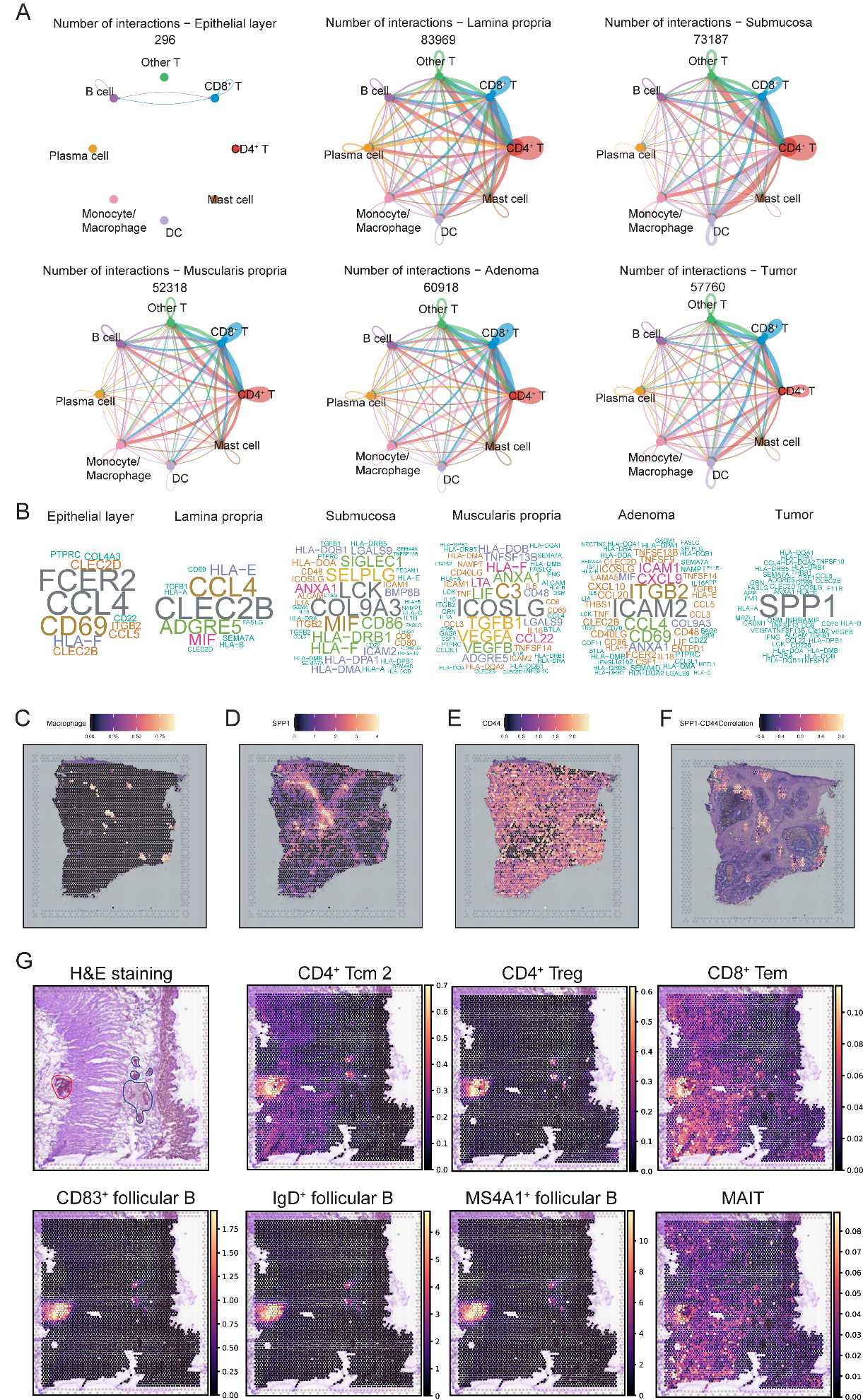


**Figure S5.** **(A)** The number of interaction and interaction strength between major cell types. **(B)** Major ligands and receptors in each site. **(C)** Spatial feature plots showing cell abundance of macrophage in the CRC slice. **(D & E)** The expression level of SPP1 (D) or CD44 (E) in the CRC slice. **(F)** Spatial feature plots demonstrating the Pearson correlation coefficient between expression of SPP1 as well as CD44 and abundance of macrophages. **(G)** Spatial plots of deconvoluted cellular subtype abundance. H & E images were from selected area of tissue section including tumor and TLS, highlighted by blue line and red line, respectively.


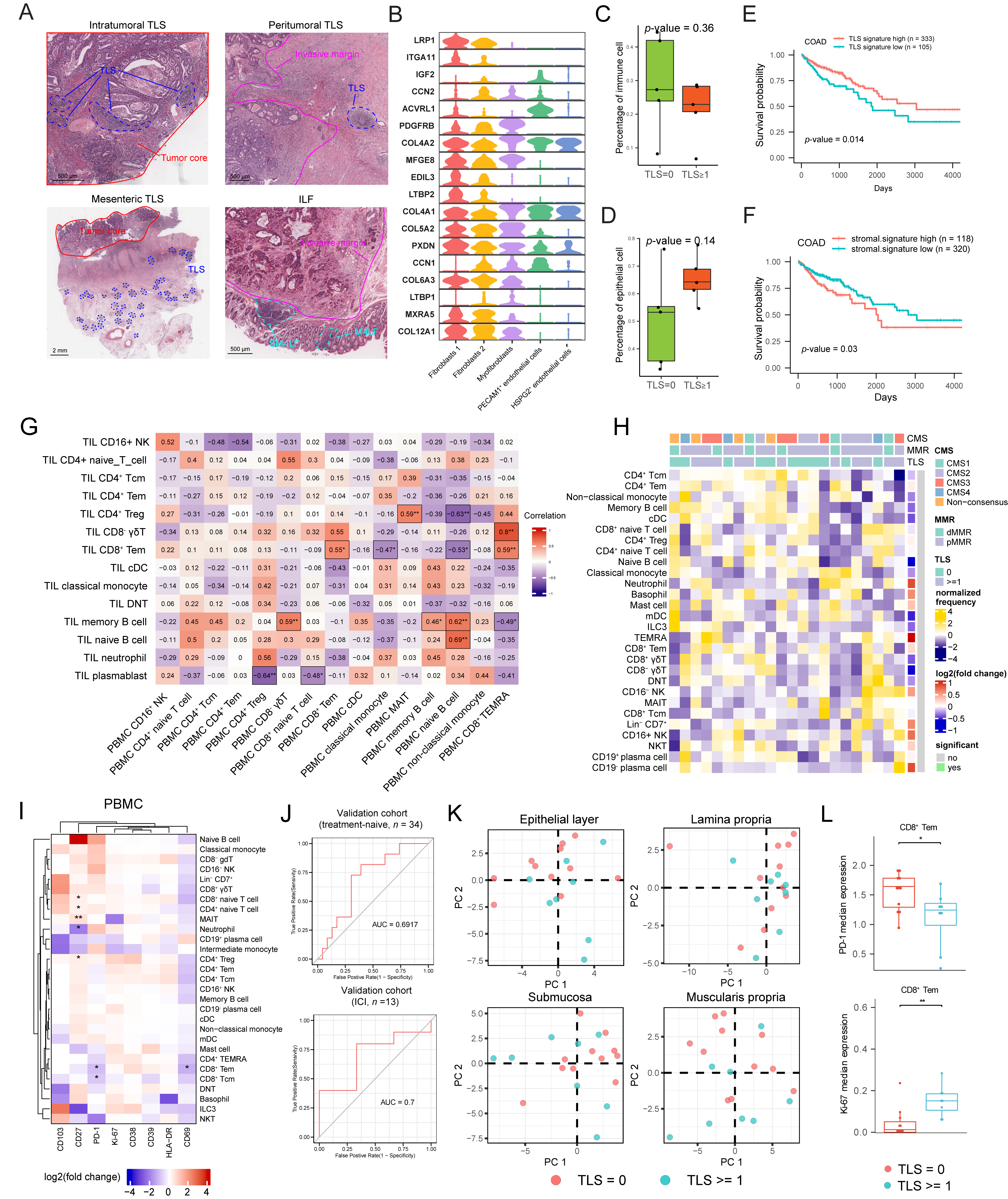


**Figure S6.** **(A)** H&E images showed TLS in the CRC macroenvironment. **(B)** Violin plots visualizing the expression of marker genes for all 5 stromal populations. **(C & D)** Bar plot showing the different percentage of immune cells (C) or epithelial cells (D) in tumor slices from patients with or without TLS. n = 5 per condition. T-test were performed. **(E)** Kaplan–Meier curves illustrating the OS for patients stratified by high and low expression of TLS signature from TCGA COAD RNA-seq cohort. A two-sided log-rank test was performed. **(F)** Kaplan–Meier curves of OS stratified by high and low expression of stromal signature based on TCGA COAD RNA-seq cohort. **(G)** Heat map showing Pearson correlation coefficient between clusters of PBMC and TIL. **p* < 0.05; ***p* < 0.01. *p* values were adjusted by Benjamini-Hochberg procedure. **(H)** Heat map showing frequency of each cluster in PBMC for patients with or without TLS. Arcsine transform was performed for frequency. (left). Log2 fold-change of proportion and significance were aligned at right side. *, *p* < 0.05; **, *p* < 0.01, likelihood ratio test. *p* values were adjusted by Benjamini-Hochberg procedure. **(I)** Comparing protein expression of each cell cluster in PBMC between patients with or without TLS. T-test was performed and *p* values were adjusted by Benjamini-Hochberg procedure. **(J)** ROC-AUC of expression of CD69 and PD-1 in classifying patients with or without TLS. Blood sample was drawn before surgery when patients with ICI were treated with Toripalimab for 12 weeks before surgery. **(K)** Principle-component analysis of cell type. Dot plots demonstrating the distribution of samples from different sites. The color of points indicated group with or without TLS. **(L)** Comparing the PD-1 and Ki67 expression of CD8^+^ Tem in muscularis propria between group with and without TLS. T-test was performed and *p* values were adjusted by Benjamini-Hochberg procedure.


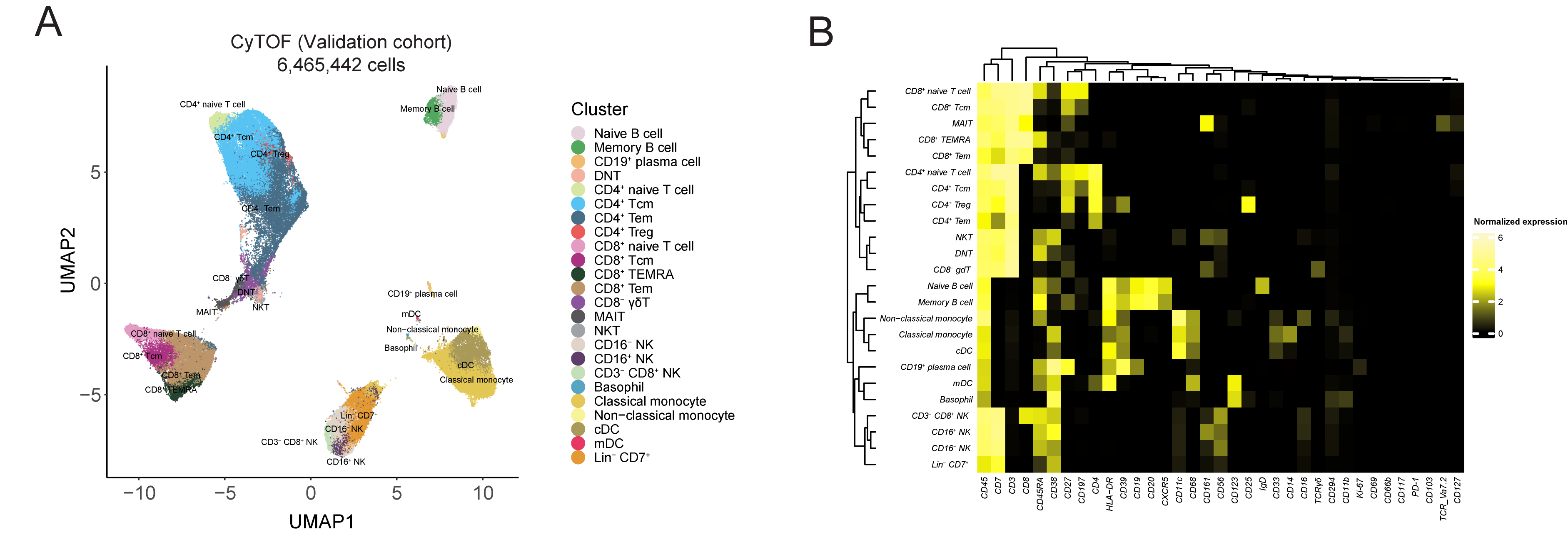


**Figure S7.** **(A)** UMAP analysis of CyTOF data from validation cohort. (B) Heat map of CyTOF panels illustrating normalized expression of markers that defined 24 clusters.


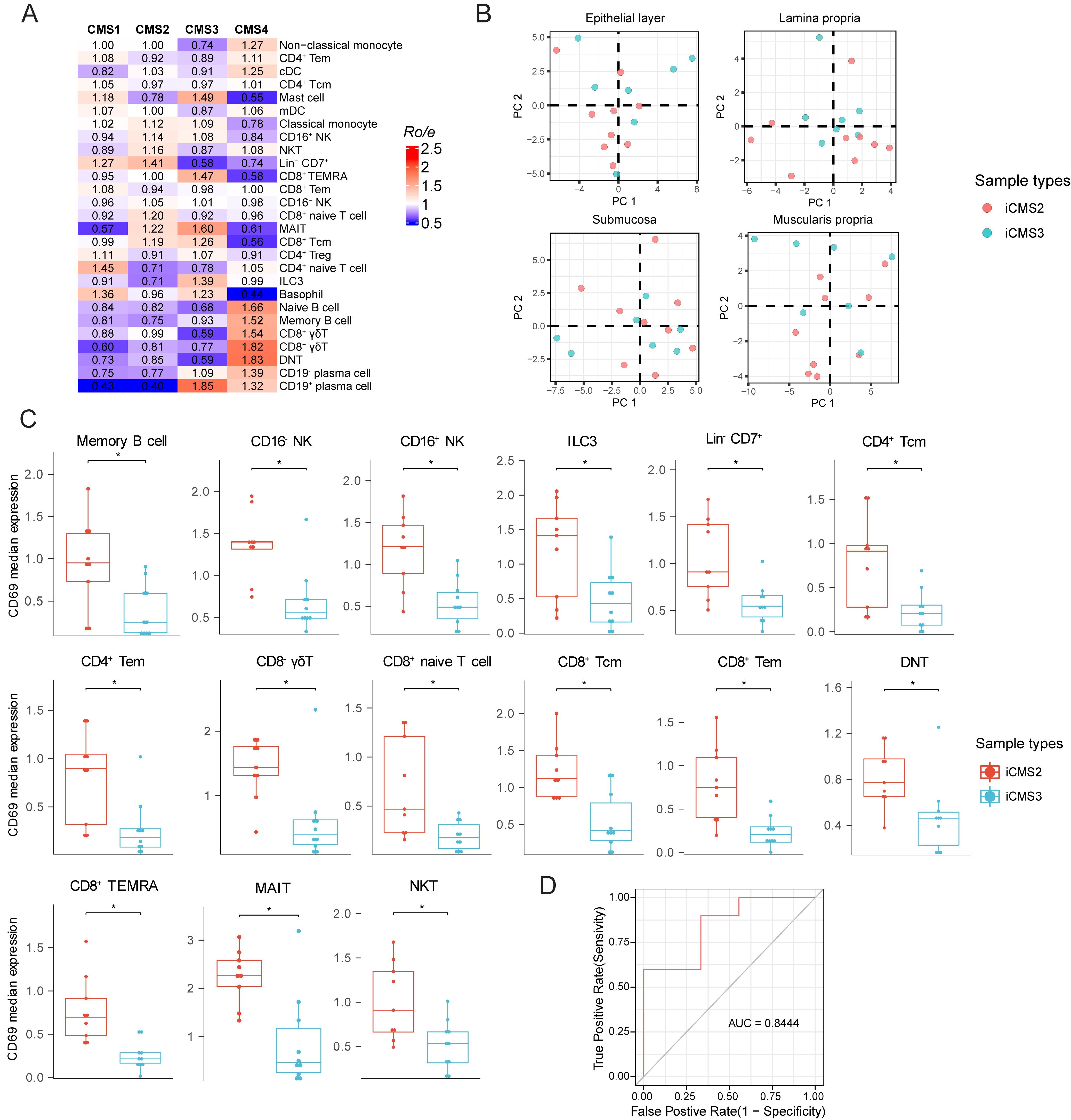


**Figure S8.** **(A)** Preference of each cluster for PBMC samples grouped by CMS. *Ro/e*, the ratio of observed to expected cell number. **(B)** Dot plots composition showing the distribution of samples from different sites. The color of points indicated tissues from patients subclassified by iCMS. **(C)** Comparing the CD69 expression of cellular populations in PBMC between groups subclassified by iCMS. T-test was performed and *p* values were adjusted by Benjamini-Hochberg procedure. **(D)** The ROC curves for the expression of CD69 in CD45^+^ cells to distinguish iCMS2 and iCMS3.


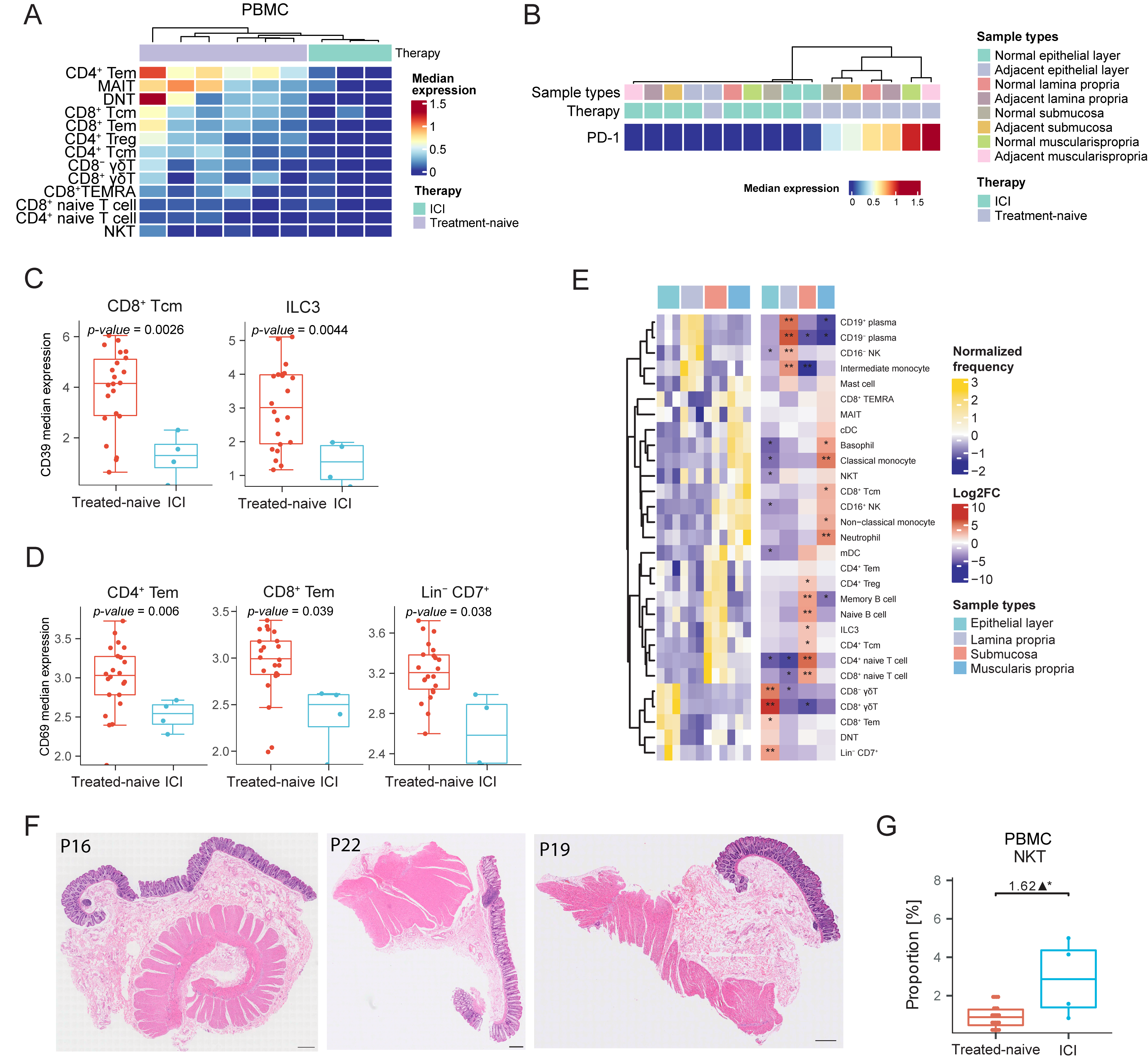


**Figure S9.** **(A)** Median PD-1 expression of T cell subtypes from PBMC in dMMR patients with or without ICI treatment. **(B)** Heat map showing the expression of PD-1 in cells from different sites for patients with or without ICI. **(C & D)** Box plots showing CD39 (C) and CD69 (D) expression of cellular populations in PBMC between patients with and without ICI. T-test was performed and *p* values were adjusted by Benjamini-Hochberg procedure. **(E)** Heat map showing the proportion of 29 clusters by Arcsine transform for 12 samples of normal bowel in patients with anti-PD-1 therapy (left) and log2 fold-change of proportion for each cluster in each sample type (right). *, *p* < 0.05; **, *p* < 0.01, likelihood ratio test. *p* values were adjusted by Benjamini-Hochberg procedure. **(F)** H & E images of bowel tissue sections from 3 patients with ICI. Scale bars = 500 μm. **(G)** Comparing the proportion of NKT cells in PBMC between CRC patients with and without ICI (patients without ICI: *n* = 19; patients with ICI: *n* = 4; number above bracket, log2 fold-change; ▲increased; ▼decreased; **P* < 0.05; ***P* < 0.01, likelihood ratio test. *P* values were adjusted by Benjamini-Hochberg procedure).


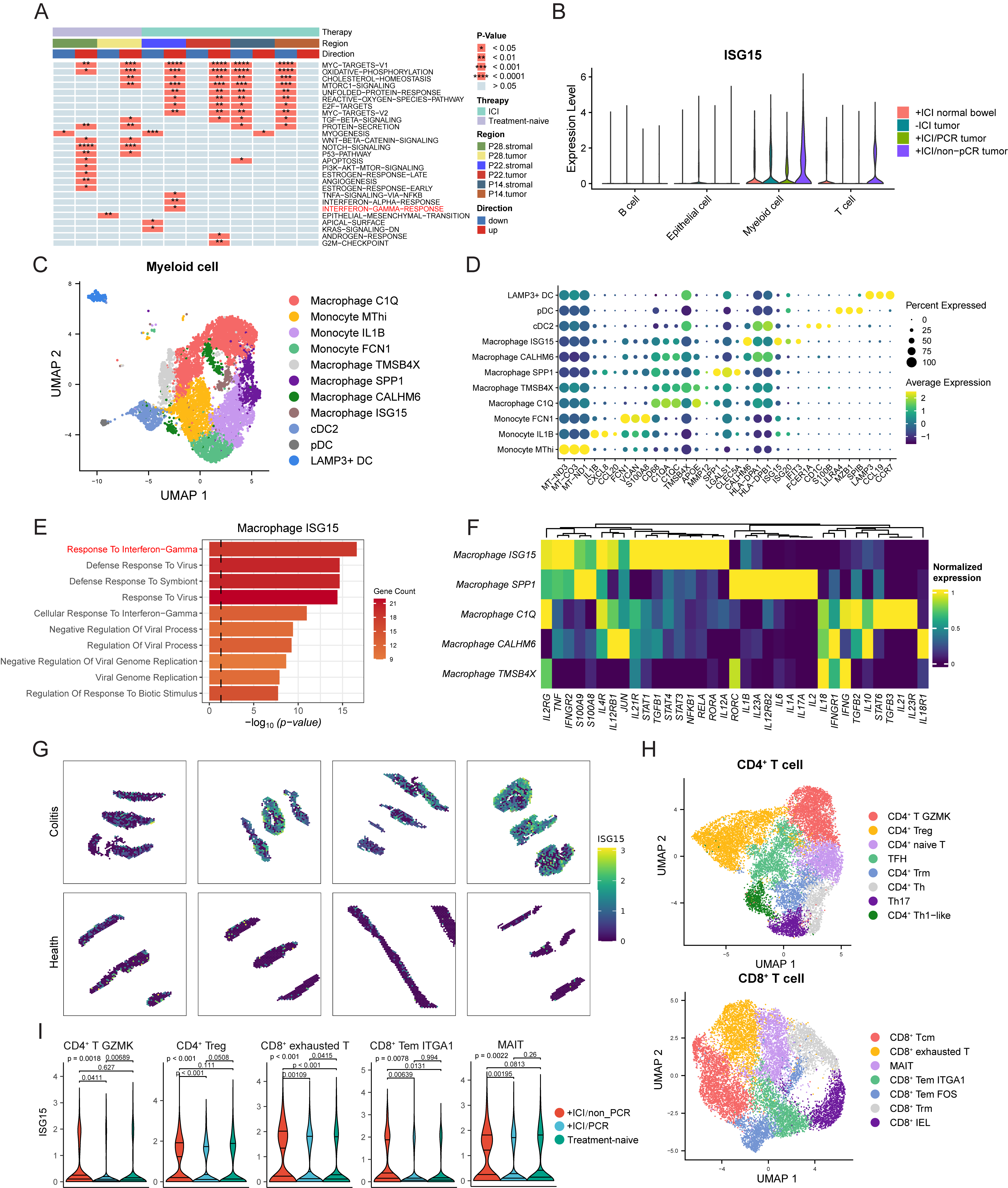


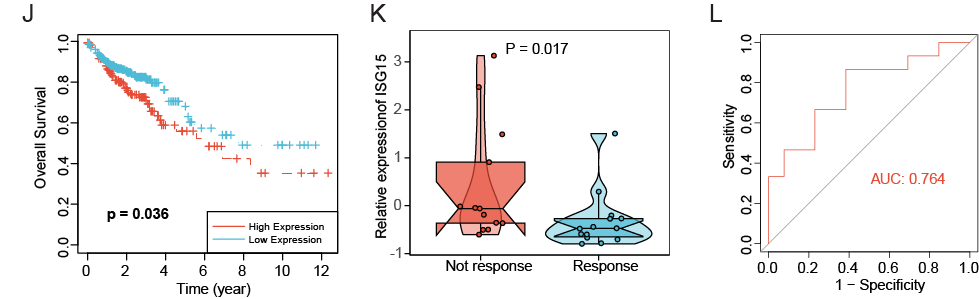


**Figure S10.** **(A)** GSEA analysis of ST data. Stromal regions and tumor cell regions from 3 patients were used. **(B)** Violin plots of ISG15 expression in broad cell types from sample groups. **(C)** UMAP representation of myeloid cell populations based on scRNA-seq data. Cells were colored by cell types. **(D)** The fraction of expressing cells (dot size) and mean expression levels (dot color) of known markers in indicated cell clusters. **(E)** Gene Ontology analysis of ISG15^+^ macrophage. Top 10 pathways were shown. **(F)** Heat map demonstrating normalized expression of inflammation-associated genes for each cluster of macrophages. **(G)** Shown was expression of ISG15 in slices of healthy bowel samples and colitis samples. **(H)** UMAPs of cells colored by CD4^+^ or CD8^+^ T cell subsets. **(I)** Violin plots of ISG15 expression in T cell subsets from sample groups. **(J)** Kaplan–Meier curves illustrating the OS for patients from the TCGA–COAD cohort stratified by high and low expression of ISG15. A two-sided log-rank test was performed. **(K)** Box plot depicting the relative expression of ISG15 between patients with response or without response. **(L)** Receiver operator characteristic curves for the expression of ISG15 between response and no response groups. AUC, area under the curve.
